# Conjugates of Heme and Intrinsically Disordered Peptides toward the Development of Synthetic Artificial Oxygen Carriers

**DOI:** 10.1021/acs.bioconjchem.6c00231

**Published:** 2026-06-29

**Authors:** Yuchen Qiao, Jeffrey Wang, Matthew Chu, Chi-Kwong Chang, Myeonggon Park, Bing Xu

**Affiliations:** † Department of Chemistry, 8244 Brandeis University, 415 South Street, Waltham, Massachusetts 02453, United States; ‡ Department of Chemistry, Michigan State University, 426 Auditorium Road, East Lansing, Michigan 48824, United States; § Martin A. Fisher School of Physics, 8244 Brandeis University, Waltham, Massachusetts 02453, United States

## Abstract

Artificial oxygen carriers (AOCs) remain an unmet medical need in clinical practice. While hemoglobin is relatively safe within red blood cells, its release into circulation causes complications due to hemoglobin degradation forming toxic hemin. Here we report the design and synthesis of a novel heme–intrinsically disordered peptide (Heme–IDP) conjugate as a potential prototype for next-generation AOCs. Heme–IDP demonstrates markedly reduced cytotoxicity compared with free hemin and protoporphyrin IX (PPIX). Fluorescence imaging further indicates that negatively charged IDP significantly reduces PPIX cellular uptake, contributing to improved biocompatibility of Heme–IDP. The use of d-enantiomeric IDP sequences enhances peptide stability. Spectroscopic analyses confirm that Heme–IDP retains the ability for axial binding. These results highlight the potential of d-peptide-based IDP to mitigate the intrinsic toxicity associated with heme derivatives, offering a promising new direction for the development of safer and more biocompatible AOCs.

## Graphical abstract

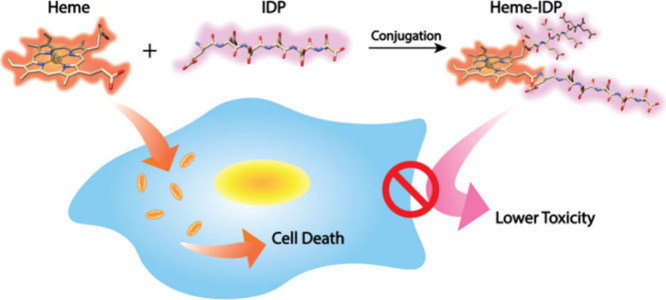

This communication reports the development of potential artificial oxygen carriers (AOCs) based on conjugates of heme and intrinsically disordered peptides (Heme–IDP). Several unmet clinical challenges drive the need for innovative AOCs, including the persistent shortage of donor blood, the risk of transfusion-related infections, and the short shelf life of stored red blood cells (RBCs). These limitations create unmet need for oxygen-carrying systems that can be stored long-term, deployed rapidly, and function effectively without the constraints of conventional blood products. To overcome these challenges, researchers have pursued multiple strategies to enhance AOC performance, such as PEGylating particle surfaces to block protein adsorption and macrophage uptake,^1^ cross-linking peptides and polymers to form protective shells,^2,3^ and encapsulating hemoglobin in zeolitic imidazolate frameworks.^4^ Among these approaches, liposomal hemoglobin vesicles (HbVs) have emerged as one of the most versatile and explored AOC platform. HbVs improve biocompatibility, allow researchers to tune oxygen affinity, and shield hemoglobin from degradation and toxicity.^5–7^ Advances in high-yield encapsulation, such as rotation–revolution mixing, have further accelerated their clinical translation.^5^ HbVs have demonstrated efficacy not only as blood substitutes, but also in targeted therapeutic applications, including preventing arrhythmias in hemorrhagic shock,^8–11^ enhancing tumor oxygenation,^12^ accelerating wound healing in diabetic models,^13^ and rescuing placental hypoxia in pre-eclampsia.^14^ In addition, researchers have also developed carbonyl-HbVs and met-HbVs for anti-inflammatory, antioxidative, and antidote functions.^15,16^

AOCs have also proven valuable beyond transfusion medicine. They have demonstrated utility in organ preservation,^17^ as perfusates for transplanted tissues,^18^ in decompression illness therapy,^19^ and as delivery systems for therapeutic gases like carbon monoxide.^16^ Perfluorocarbon-based formulations, including albumin-derived perfluorocarbon-based oxygen carriers (PFOCs), also show promise in preventing hypoxic tissue damage during extreme hemodilution.^20–22^ Moreover, hybrid AOCs incorporating nanozymes can actively scavenge reactive oxygen species, offering prolonged protection against oxidative stress.^23^ These diverse applications underscore the growing recognition: AOCs are not merely blood substitutes, but multifunctional therapeutic tools with potentials to address a wide range of hypoxia-related and oxidative stress-associated conditions.

However, even the most advanced hemoglobin-based oxygen carriers (HBOCs) and PFOCs still face significant challenges, including toxicity, immune activation, poor circulate persistence, and limited biocompatibility.^5,24,25^ One of the major concerns with circulating hemoglobin is that its degradation can generate toxic hemin. Hemin forms when hemoglobin releases heme, which then becomes oxidized to the ferric state. Hemin is highly cytotoxic, as it disrupts cellular membranes, induces oxidative stress, and activates immune receptors such as TLR4.^26–30^ Therefore, the continued development of novel AOC materials with enhanced stability, biocompatibility, and targeted functionalities is essential to realize their full clinical potential. To address this need, we aim to explore heme–IDP conjugates for AOC applications, with the primary objective of employing IDPs to minimize both the formation and toxicity of hemin.

We choose heme-based materials over PFOCs for several reasons: (i) Heme binds oxygen chemically rather than relying on physical dissolution. (ii) The Fe^2+^ center in the heme’s porphyrin ring binds O_2_ cooperatively and releases it in response to local tissue hypoxia, closely mimicking red blood cell physiology. (iii) Chemical modification can precisely tune the oxygen affinity (*P*_50_) of heme-based carriers,^31–33^ enabling controlled oxygen unloading exactly where it is needed. However, free heme is hydrophobic, and uncontrolled cellular uptake of free heme can cause oxidative damage, inflammation, and cytotoxicity. To address these shortcomings, we conjugate hydrophilic IDPs to heme to generate Heme–IDPs. IDPs offer several advantages: they are highly flexible, lack stable secondary structure, and present a dense array of hydrophilic side chains, which can improve aqueous solubility and sterically shield the heme from unwanted interactions. Our recent study demonstrates that increasing IDP density significantly reduces nonspecific protein adsorption, which in turn minimizes unwanted binding to plasma proteins.^34^ This feature suggests that IDPs should lower cellular uptake. To further enhance the stability of the Heme–IDP, we choose d-enantiomeric IDPs. This dual effect both enhances biocompatibility and reduces hemin-mediated toxicity, making Heme–IDP conjugates a promising platform for next-generation AOCs. Thus, the objective of this study is to examine the cytotoxicity of Heme–IDP.

Based on the above rationale, we employed solid-phase peptide synthesis to prepare Heme–IDPs designed with d-enantiomeric IDP sequences to enhance structural stability and resistance to proteolytic degradation. This molecular design aims to emulate the oxygen-binding functionality of hemoproteins while minimizing the cytotoxic effects typically associated with free heme or its degradation products. Remarkably, cell viability assays revealed that Heme–IDP exhibits substantially lower cytotoxicity compared to free hemin and protoporphyrin IX (PPIX). Fluorescence imaging further demonstrated that PPIX–IDP shows limited cellular internalization, which likely contributes to the enhanced biocompatibility of Heme–IDP. Spectroscopic analyses confirmed that Heme–IDP retains the capacity for axial binding of oxygen, suggesting its potential as an AOC. Collectively, these results establish Heme–IDP as a new AOC candidate that leverages the unique flexibility and biocompatibility of IDPs to overcome long-standing toxicity challenges in heme-based oxygen carrier design (Scheme 1).

**Scheme 1:**
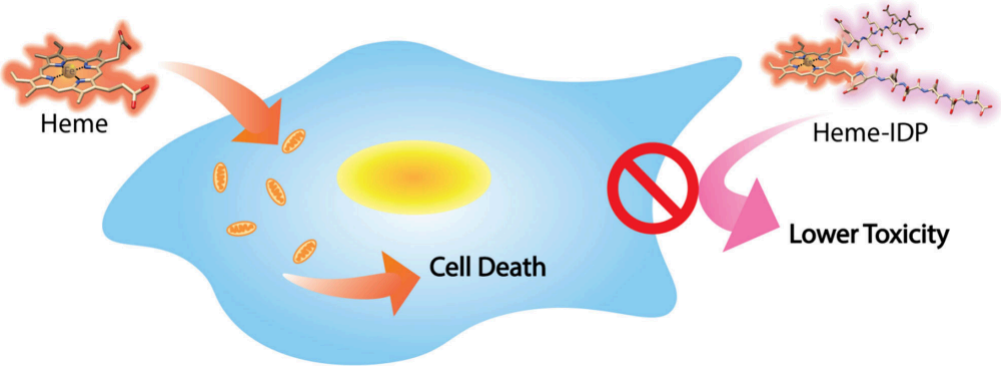
Schematic Illustration Showing That Heme–IDP Exhibits Reduced Cytotoxicity Compared to Heme

We selected the d-hexaglutamate sequence (e_6_) as a representative IDP motif since a variety of proteins contain l-hexaglutamate sequence in the intrinsically disordered regions.^35–37^ Similar acidic IDP segments, when conjugated to aromatic cores, have previously shown disordered behaviors even when forming assemblies.^38,39^ Building on these findings, we coupled the negatively charged peptide to protoporphyrin IX (PPIX) and heme (HM), the iron-containing porphyrin central to hemoglobin. Conjugation of d-glutamate peptides yielded PPIX–(e_6_)_2_ and HM–(e_6_)_2_ (Figure 1A), while the corresponding l-glutamate analogues, PPIX–(E_6_)_2_ and HM–(E_6_)_2_, were synthesized as stereochemical controls (Figures S1–S5).

**Figure 1:**
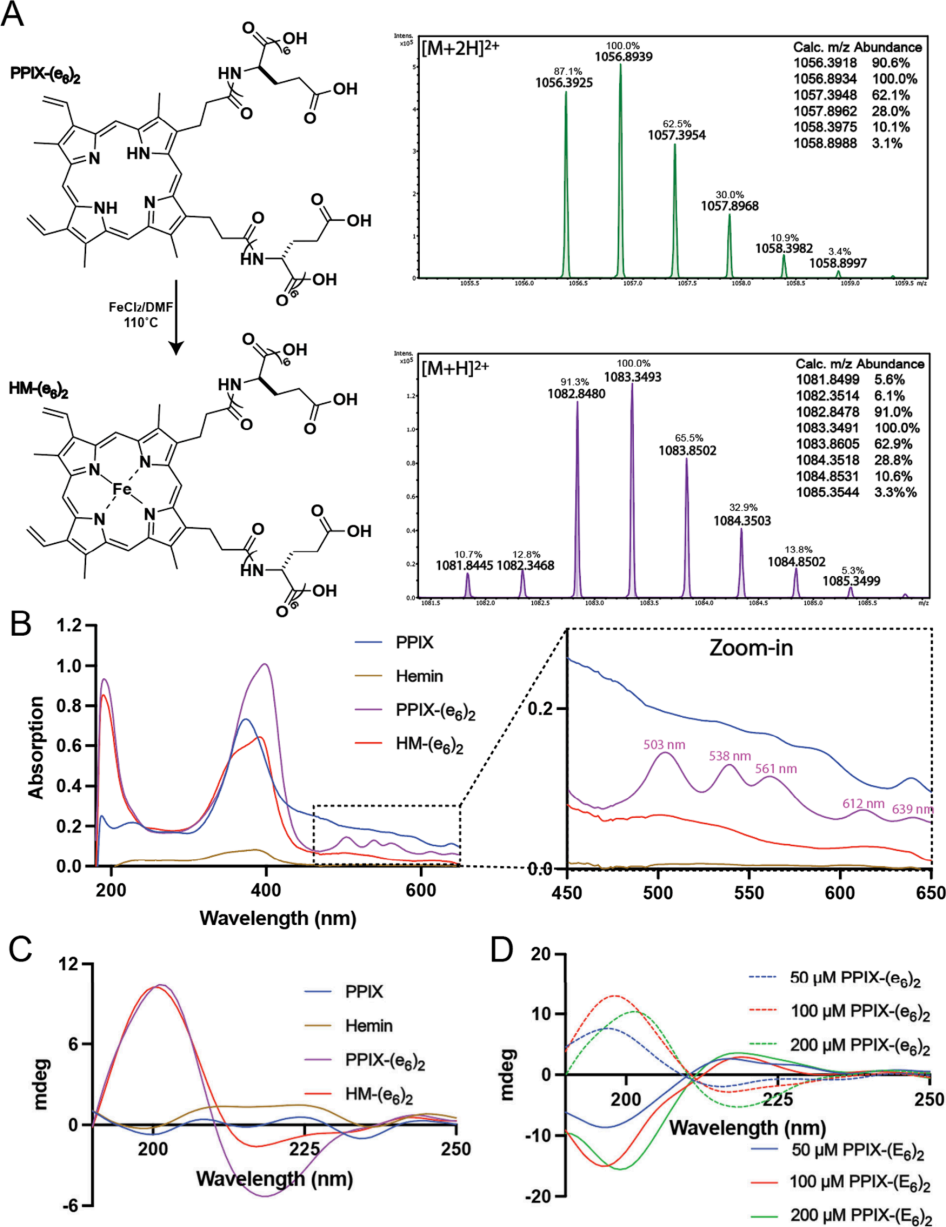
(A) Molecular design, synthetic route, and MS spectra of PPIX–(e_6_)_2_ and HM–(e_6_)_2_. (B) UV–vis absorption spectra and (C) CD spectra of 200 μM PPIX, hemin, PPIX–(e_6_)_2_, and HM–(e_6_)_2_. (D) CD spectra of PPIX–(e_6_)_2_ and PPIX–(E_6_)_2_ at 50, 100, and 200 μM. All samples were dissolved in water at pH 7.

We synthesized the PPIX–IDP conjugates by solid-phase peptide synthesis (SPPS). Using the PPIX–IDPs as precursors for direct metal insertion (Scheme S1), we obtained the corresponding HM–IDP analogues. The high-resolution mass spectrum (HRMS) of HM–(e_6_)_2_ displayed the expected iron-associated isotopic pattern, confirming successful coordination of the porphyrin with Fe (Figure 1A).

To examine the photophysical properties of these conjugates, we compared them with commercially available PPIX and hemin and recorded fluorescence, UV–vis, and CD spectra. PPIX–(e_6_)_2_ exhibited the characteristic Soret band at 400 nm and well-defined Q bands between 500–650 nm, whereas HM–(e_6_)_2_ retained a strong Soret band but showed minimal Q-band features, consistent with iron coordination suppressing these electronic transitions^40^ (Figure 1B). The absorption intensity of PPIX–(e_6_)_2_ increased proportionally with concentration, with both the Soret and Q bands becoming stronger from 50 to 200 μM (Figure S6). At 500 μM, the Soret band broadened, consistent with concentration-dependent aggregation. Notably, unconjugated PPIX tends to aggregate in water due to its hydrophobic planar macrocycle, which promotes π–π stacking and leads to diminished or broadened Soret and Q bands^41,42^ (Figure 1B and Figure S6C). Improved aqueous solubility from the glutamate-rich peptide sharpens the spectral features of PPIX–(e_6_)_2_, with a similar effect observed for HM–(e_6_)_2_ relative to free hemin. The weaker Soret band and suppressed Q bands of hemin are consistent with Fe­(III)-induced perturbation of porphyrin electronic transitions.^43^

HM–(e_6_)_2_ and PPIX–(e_6_)_2_, both bearing d-peptide conjugates, display CD features characteristic of disordered conformations, consistent with the intrinsic disorder expected for IDP-derived sequences. In contrast, free hemin and PPIX show no discernible CD features, as they lack peptide components (Figure 1C). The CD spectra of the l-peptide conjugates PPIX–(e_6_)_2_ were mirror images of their d-peptide counterparts, confirming the expected opposite chirality, with signal intensity increasing from 50 to 100 μM and a slight red shift at 200 μM, likely due to the aggregation at higher concentrations (Figure 1D).

Fluorescence measurements further support concentration-dependent aggregation, with PPIX–(e_6_)_2_ and PPIX–(E_6_)_2_ showing increased emission from 10 to 50 μM followed by self-quenching above 100 μM, while unconjugated PPIX exhibited much weaker fluorescence consistent with poor solubility (Figure S7A–C). In contrast, HM–(e_6_)_2_ and HM–(E_6_)_2_ showed strongly quenched fluorescence relative to PPIX conjugates, paralleling the behavior of free hemin and confirming iron coordination (Figure S7D–F).

The particle size of HM–(e_6_)_2_ was characterized by DLS (Figure S8A,B), revealing a polydisperse distribution with two major populations at ∼6 nm and ∼353 nm, spanning size ranges of 1–10 nm and 100–10000 nm, respectively. TEM images show individual particles of 5 ± 1 nm along with larger aggregates ranging from tens to hundreds of nanometers, in good agreement with the DLS results (Figure S8C).

Hemin toxicity has historically limited its development as a drug candidate or oxygen carrier for clinical applications.^44,45^ Likewise, PPIX is restricted by pronounced phototoxicity.^46^ To evaluate whether conjugation to intrinsically disordered peptides (IDPs) could mitigate these limitations, we assessed the biocompatibility of the IDP–porphyrin conjugates (Figures 2 and S10). Free PPIX exhibited substantial cytotoxicity toward two mammalian cell lines (HeLa and Saos2), with GI_50_ values of 1.8 and 2.5 μM, respectively (Figure 2A,B). In contrast, PPIX–(e_6_)_2_, bearing d-glutamate peptide chains, showed markedly reduced toxicity in both cell lines, with GI_50_ values exceeding 80 μM, corresponding to more than a 30-fold decrease in cytotoxicity. Similarly, HM–(e_6_)_2_ displayed lower apparent toxicity than free hemin, indicating that IDP conjugation improves the biocompatibility of heme-based compounds.

**Figure 2:**
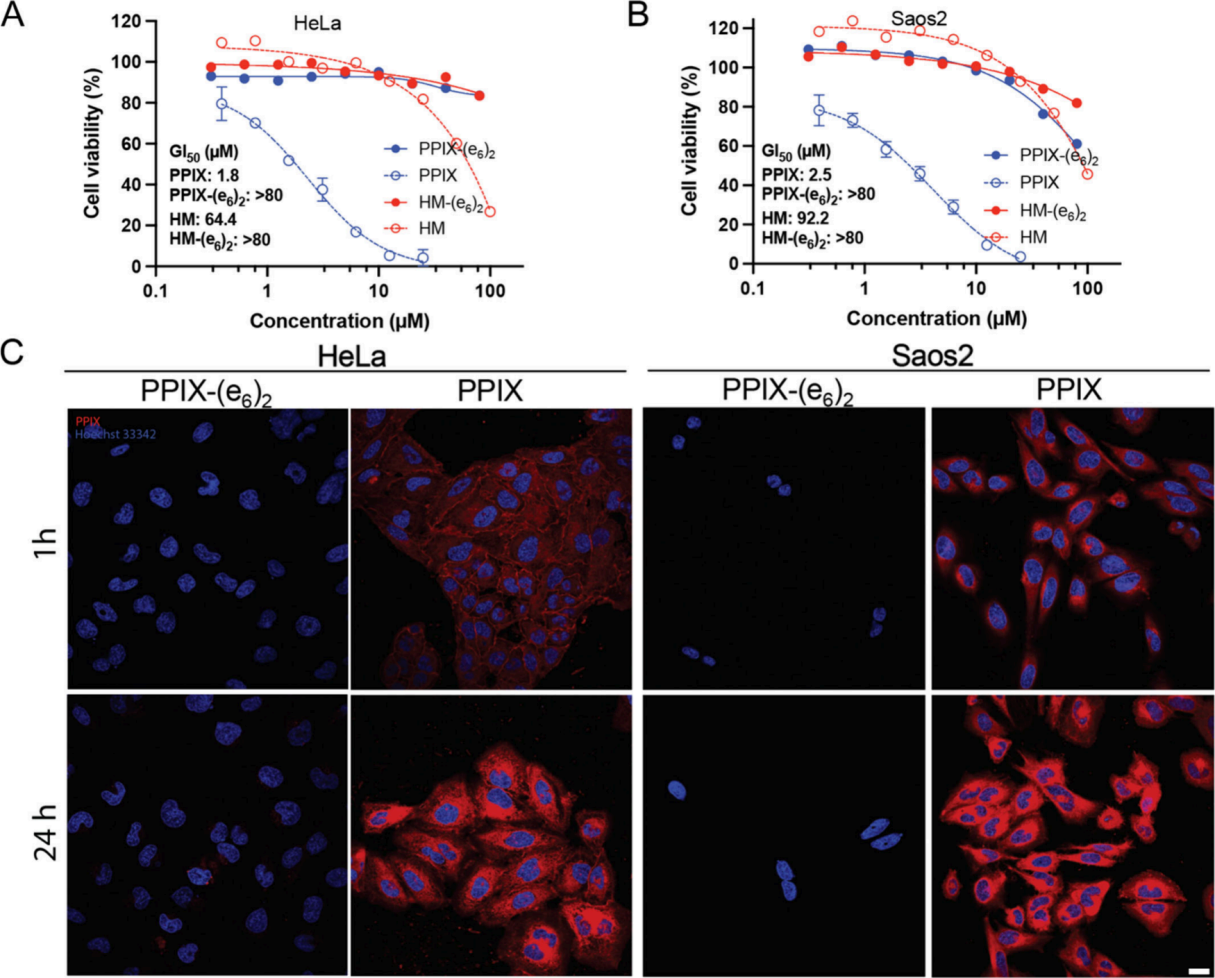
(A) Cytotoxicity of HeLa cells and (B) Saos2 cells treated with PPIX, hemin, PPIX–(e_6_)_2_, and HM–(e_6_)_2_ for 24 h. (C) Cellular uptake of 50 μM PPIX–(e_6_)_2_ and 10 μM PPIX in HeLa or Saos2 cells after 1 and 24 h of treatment. Scale bar = 20 μm.

Because both hemin and HM–IDP conjugates exhibit strongly quenched fluorescence, as established spectroscopically (Figure S7), confocal laser scanning microscopy (CLSM) analysis of HM-based compounds was not informative. Thus, to investigate intracellular localization, we performed CLSM based on the fluorescence of PPIX. Consistent with the cytotoxicity results, free PPIX showed strong intracellular fluorescence after treatment, indicating efficient cellular uptake and accumulation. This process was time dependent: minimal signal was detected after 1 h, whereas pronounced cytoplasmic accumulation was observed after 24 h in both HeLa and Saos2 cells, indicating little cell-line selectivity (Figure 2C). In contrast, PPIX–(e_6_)_2_ displayed minimal intracellular fluorescence even after 24 h of incubation, consistent with its low cytotoxicity and limited cellular internalization (Figure 2C). Time-lapse CLSM imaging of HeLa cells treated with PPIX–(e_6_)_2_ for 1 h further confirmed this behavior (Figure S11). Although diffuse fluorescence was observed in the extracellular medium, the compound preferentially formed aggregates outside the cells rather than being internalized. These aggregates appeared as bright, size-variable puncta in the extracellular space, while the cells remained largely impermeable to the IDP-conjugated PPIX.

Although the l-IDP conjugate PPIX–(E_6_)_2_ showed reduced cellular uptake compared to free PPIX (Figure S10), with behavior comparable to its d-peptide counterpart PPIX–(e_6_)_2_, the MTT assay revealed slightly higher cytotoxicity for the l-IDP conjugate. Specifically, PPIX–(E_6_)_2_ exhibited GI_50_ values of 76 μM in HeLa cells and 67 μM in Saos2 cells after 2 days of treatment (Figure S10). This modest increase in toxicity may arise from the susceptibility of l-amino acid sequences to proteolysis in cellular environments, which could gradually release PPIX-containing fragments.

To test this hypothesis, we performed an in vitro protease digestion assay. We incubated PPIX–(E_6_)_2_ and PPIX–(e_6_)_2_ with proteinase K, a broad-spectrum protease,^47^ in neutral aqueous solution and quenched the reactions at defined time points for LC–MS (Figures S12–S16). We used PPIX-based conjugates instead of HM–IDPs because, under protease-compatible aqueous conditions, HM conjugates formed multiple coordination species and aggregates that complicated chromatographic separation and peak assignment (Figure S9). LC–MS analysis showed that PPIX–(e_6_)_2_ remained intact throughout the experiment, consistent with the expected protease resistance of d-peptides (Figures 3A and S16). In contrast, proteinase K rapidly digested PPIX–(E_6_)_2_, with near-complete cleavage within 30 min (Figure 3B). MS indicated a stepwise cleavage: PPIX–(E_6_)_2_ (i.e., PPIX–E_12_) first converted to PPIX–E_10_ and then to PPIX–E_8_ (Figures S12–S15). These results suggest that proteinase K cleaves both peptide chains in parallel, sequentially shortening each E_6_ segment. The absence of species shorter than PPIX–E_8_, between 30 min and 2 h, indicates reduced accessibility or increased stability of the shorter peptide fragments.

**Figure 3:**
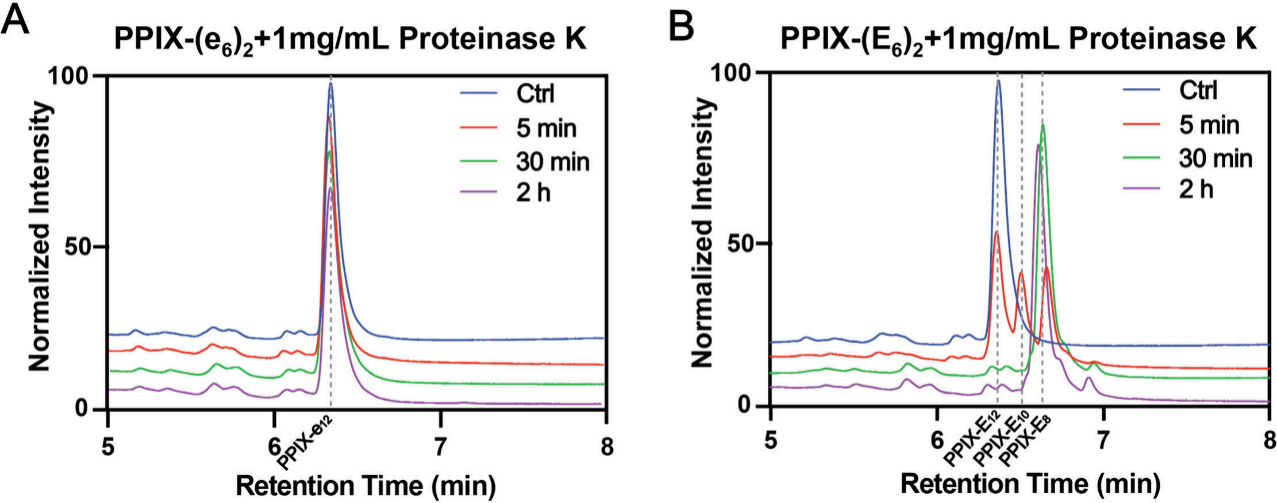
LC chromatograms at 400 nm of 200 μM (A) PPIX–(e_6_)_2_ and (B) PPIX–(E_6_)_2_ before treatment (control, blue) and after incubation with 1 mg/mL proteinase K for 5 min (red), 30 min (green), and 2 h (purple) at 37 °C in water at pH 7. Gray dashed lines indicate the corresponding cleavage products for each peak, as verified by LC–MS (see the Supporting Information).

A well-established approach to evaluate axial oxygen binding in heme and analogs is to monitor characteristic shifts in the UV–vis absorption spectrum.^48^ Although the hexaglutamate peptide significantly improves solubility in water, the iron-associated features of HM–(e_6_)_2_ remain poorly resolved under neutral aqueous conditions, likely due to axial coordination of hydroxide or formation of μ-oxo or hydroperoxo species at physiological pH^49–51^ (Figure 1B). LC–MS analysis of HM–(e_6_)_2_ dissolved in water revealed multiple dimeric species corresponding to [M + O] and [M + OOH] adducts, supporting the coexistence of μ-oxo- and hydroperoxo-bridged dimers under these conditions (Figure S9A). In contrast, these dimeric species were largely suppressed in pyridine upon addition of a reductant, yielding a dominant monomeric HM–(e_6_)_2_ peak (Figure S9B). To obtain clearer spectral signatures and maintain full solubility, HM–(e_6_)_2_ was dissolved in 1 M pyridine or imidazole, two ligands known to bind strongly to ferric heme and disrupt μ-oxo dimerization (Figure 4).

**Figure 4:**
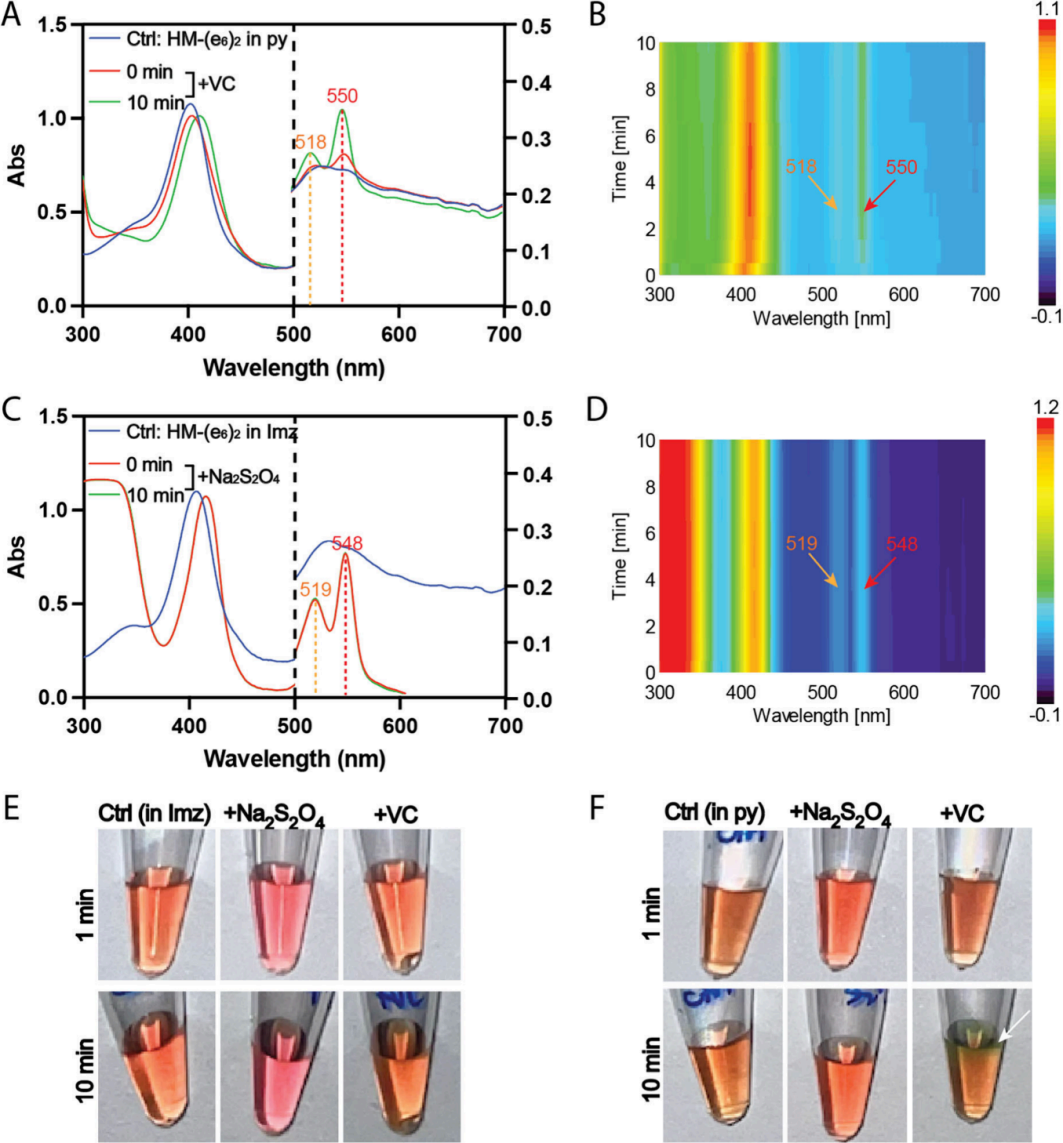
(A) UV–vis absorption spectra of 200 μM HM–(e6)_2_ in 1 M pyridine (control) and after addition of 10 mM vitamin C (VC) at 0 and 10 min. (B) Corresponding heatmap showing spectral changes over 10 min. (C) UV–vis absorption spectra of 200 μM HM–(e6)_2_ in 1 M imidazole (control) and after addition of 10 mM Na_2_S_2_O_4_ at 0 and 10 min. (D) Corresponding heatmap showing spectral changes over 10 min. (E) Photographs of 200 μM HM–(e6)_2_ in 1 M imidazole (control) and after treatment with 10 mM Na_2_S_2_O_4_ or vitamin C for 1 and 10 min. (F) Photographs of 200 μM HM–(e6)_2_ in 1 M pyridine (control) and after treatment with 10 mM Na_2_S_2_O_4_ or vitamin C for 1 and 10 min. The white arrow indicates the greenish layer formed at the top of the solution.

To mimic a reducing environment relevant to biological systems, we used vitamin C (VC) to convert Fe­(III) to Fe­(II). In 1 M pyridine, HM–(e_6_)_2_ initially showed a broad, unresolved absorption feature between 520–550 nm; however, upon VC addition, two distinct bands corresponding to the Q_v_/Q_0_ (β/α) transitions emerged immediately (0 min) and stabilized at 518 and 550 nm (Figure 4A) under ambient conditions. This clearly indicates formation of the Fe­(II) species, which exhibits well-resolved oxygen on/off binding features.^52,53^ A corresponding heatmap recorded over 10 min traced the emergence and stabilization of the two peaks, reaching steady-state intensity within approximately 2 min (Figure 4B).

In contrast, the same two-peak pattern did not emerge in imidazole-containing solution upon VC treatment (Figure S17C,D), suggesting that free imidazole weakly binds to the heme center and that VC alone is insufficient to fully reduce Fe­(III) under these conditions. Therefore, we evaluated sodium dithionite (Na_2_S_2_O_4_), a strong and widely used reductant for heme complexes.^54^ Upon addition of Na_2_S_2_O_4_, two well-resolved peaks at 519 and 548 nm appeared immediately in both imidazole (Figure 4C) and pyridine solutions (Figure S17A), and their intensities remained stable over time (Figures 4D and S17B), confirming robust formation of the reduced Fe­(II) state.

The absorption spectrum of free hemin subjected to the same reductive conditions used for HM–(e_6_)_2_ was also examined (Figure S18). In 1 M pyridine, ferric hemin displayed a broad, intense band near 400 nm along with unresolved, low-intensity features between 500 and 600 nm, closely resembling the spectrum of HM–(e_6_)_2_ in the same solvent. Upon addition of reductants, distinct α and β peaks emerged in the 500–600 nm region. Reduction with vitamin C (VC) produced these peaks gradually, accompanied by a slight red shift over 10 min (Figure S18A,B), whereas sodium dithionite yielded a well-resolved two-peak pattern immediately, with minimal change over time (Figure S18C,D).

In imidazole solution, however, ferric hemin showed split Soret features around 400 nm, a behavior commonly attributed to mixed coordination states and partial aggregation.^55,56^ Addition of VC did not alter this spectral pattern (Figure S18E,F), mirroring the behavior observed for HM–(e_6_)_2_ in imidazole under VC treatment (Figure S17C,D). In contrast, sodium dithionite collapsed the split Soret features into a single peak at 410 nm and induced the gradual development of a shoulder near 450 nm. The α and β peaks at 500–600 nm also became more pronounced upon reduction, though their relative intensity remained lower than in pyridine, reflecting differences in ligand strength and coordination equilibrium.

Visual inspection of the reaction tubes confirms the evolution of the system. When dissolved in imidazole, HM–(e_6_)_2_ formed a transparent orange–red solution. Upon addition of Na_2_S_2_O_4_, the solution immediately turned pinkish red and remained essentially unchanged over 10 min. In contrast, addition of VC caused little to no visible color change, either immediately or after 10 min (Figure 4E). In pyridine containing solution, HM–(e_6_)_2_ initially appeared light brown–orange. Reduction with Na_2_S_2_O_4_ produced a warm orange-red color that remained stable over 10 min. However, reduction with VC hardly enhances the red coloration; instead, after approximately 10 min, a distinct greenish layer developed at the top of the solution (Figure 4F). This behavior is attributed to oxygen-dependent oxidative transformation of the heme center at the air–liquid interface, leading to formation of oxidized heme intermediates, most plausibly verdoheme-like species.^57,58^ A nearly identical color transition for free hemin under the same conditions confirms that the observed behavior originates from the heme motif rather than peptide conjugation (Figures S19 and S20). Overall, these results demonstrate that conjugation of heme to an IDP preserves the fundamental redox and ligand-binding behavior of the heme center, while offering a viable strategy to modulate its oxygen-carrying properties.

In summary, we show that conjugation of PPIX and heme to glutamate-rich IDP sequences effectively modulate the biological and physicochemical behavior of these porphyrins. Prior studies have shown that a hydrophobic environment, as in picket-fence porphyrins, is essential for stabilizing oxygen binding, yet the short lifetime of the oxygen-bound state remains a key challenge.^59–61^ In this work, we do not aim to optimize oxygen binding, but instead introduce a heme–IDP conjugation strategy that improves biocompatibility while preserving heme activity, providing a potential platform for future designs incorporating more defined hydrophobic environments. IDP attachment enhances aqueous solubility, suppresses uncontrolled aggregation, and preserves the intrinsic photophysical signatures of the porphyrin core. This approach should be applicable to more sophisticated porphyrin architectures, such as porphyrin cages^62,63^ and films,^64,65^ and to broader applications, such as photodynamic therapy.^66,67^ Biological studies show that IDP conjugation substantially reduces the cytotoxicity of both PPIX and hemin, consistent with diminished cellular uptake and extracellular aggregation. Comparative analysis of d- and l-peptide conjugates reveals that d-peptide conjugates resist enzymatic degradation more effectively, while l-IDP conjugates undergo rapid proteolysis. Although excess pyridine or imidazole in aqueous solution can axially coordinate to the iron center and compete with molecular oxygen binding, the preserved heme reactivity, especially axial oxygen binding ability, provides a useful platform for further molecular engineering. The next step is to introduce a single intramolecular axial pyridine or imidazole ligand into the heme–IDP scaffold to better replicate the native coordination site and enable controlled oxygen binding, as demonstrated in hemoglobin mimics.^68–70^ Overall, IDP conjugation offers a modular strategy to improve solubility, regulate aggregation, reduce cytotoxicity, and maintain essential heme chemistry, thus providing a starting point to advance the design of porphyrin-based oxygen carriers and bioinspired materials.

## Supplementary Material



## Notes

Detailed experimental procedures and materials; LC–MS analyses of individual compounds and enzymatic degradation experiments; fluorescence emission and UV–vis absorption spectra; additional MTT cell viability data; and time-lapse CLSM images of cellular uptake (PDF)
