# Supplementary material for: Conjugates of Heme and Intrinsically Disordered Peptides toward the Development of Synthetic Artificial Oxygen Carriers
Source: Bioconjug Chem. 2026 Jun 29;37(7):1341–9. doi: 10.1021/acs.bioconjchem.6c00231 (PMC13377603; doi:10.1021/acs.bioconjchem.6c00231)
Supplement: Supplementary file 1 [file bc6c00231_si_001.pdf]

Supporting information for

The Conjugates of Heme and Intrinsically Disordered Peptides toward the Development of Synthetic Artificial Oxygen Carriers

Yuchen Qiao[1], Jeffrey Wang[1], Matthew Chu[1], Chi-Kwong Chang[2], Myeonggon Park[3], Bing Xu[1,*]

[1]Department of Chemistry, Brandeis University, 415 South St., Waltham, MA 02453, USA

[2]Department of Chemistry, Michigan State University, 426 Auditorium Road, East Lansing, MI 48824, USA

[3]Martin A. Fisher School of Physics, Brandeis University, Waltham, MA 02453, USA

Table of Contents

Supplemental Experimental Procedures 3
Supporting figures 7

Supplemental Experimental Procedures

Materials

2-Cl-trityl chloride resin (1.02 mmol/g) and Fmoc protected amino acid building blocks were purchased from GL Biochem (Shanghai, China). O-(Benzotriazol-1-yl)-N,N,N′,N′-tetramethyluronium hexafluorophosphate (HBTU) was purchased from Chem impex. N, N-diisopropylethylamine (DIPEA) were purchased from TCI America. Protoporphyrin IX, pyridine and imidazole were purchased from Sigma-Aldrich. Hemin was synthesized by Dr. Chi-Kwong Chang. Anhydrous iron(II) chloride ($FeCl_2$), Sodium hydrosulfite ($Na_2S_2O_4$), L-Ascorbic acid sodium salt (VC) was purchased from Thermo Fisher Scientific. Proteinase K was purchased from Sigma-Aldrich (Proteinase K from Tritirachium album, Product Number P2308). Dimethylformamide (DMF), methylene chloride (DCM), trifluoroacetic acid (TFA), methanol (MeOH), and other reagents and solvents were purchased from Fisher Chemical. All reagents and solvents were used without further purification.

Instruments

All crude compounds were purified using a reverse phase HPLC (Agilent 1100 Series) with HPLC grade water (0.1% TFA) and HPLC grade acetonitrile (0.1% TFA) as eluents. LC-MS spectra were obtained on a Bruker timsTOF Pro Mass Spectrometer equipped with an Elute UHPLC chromatograph. CLSM images were acquired using “Dagahra” Nikon AX-R Resonant scanner. Fluorescence emission spectra were recorded using a Shimadzu RF-5301PC spectrofluorometer. Circular dichroism (CD) and UV–vis absorption spectra were collected using a Jasco J-810 spectropolarimeter.

Peptide synthesis

Synthesis of PPIX-IDPs. PPIX-IDPs were synthesized via solid-phase peptide synthesis (SPPS). 2-Cl-trityl chloride resin was dipped in methylene chloride (DCM) for 5 min, followed by loading the amino acid building blocks using N, N-diisopropylethylamine (DIPEA) in DCM overnight. A capping solution (DCM:MeOH:DIPEA = 17:2:1) was added for 30 min, and 20% piperidine in dimethylformamide (DMF) was added for another 30 min for deprotection. For subsequent couplings, Fmoc-protected amino acid building blocks, HBTU, HOBt, and DIPEA

were added in a molar ratio of 1:1:1:4 and allowed to react for 2 h, with DMF washes performed after each coupling step. After deprotection of the final amino acid, protoporphyrin IX (PPIX) was activated with 2 equiv of HBTU and 8 equiv of DIPEA and coupled overnight. Following coupling, the resin was washed with DMF and DCM. Peptides were then cleaved from the resin using trifluoroacetic acid (TFA) for 1 h. After concentration of the cleavage solution, cold diethyl ether was added to precipitate the crude peptides, which were subsequently purified by HPLC.

Synthesis of HM-IDPs. HM-IDPs were synthesized from the corresponding PPIX-IDPs (Scheme S1). Briefly, PPIX-IDPs were dissolved in DMF and treated with 50 equiv of $FeCl_2$. The reaction mixture was heated at 110 ˚C for 24 h. After completion, DMF was removed under reduced pressure. The residue was dissolved in water, and 1 M NaOH was added to adjust the solution to pH 9. Insoluble materials were removed by filtration, and the filtrate was collected. The solution was then acidified to pH 2 by addition of 1 M HCl and stirred for 2 h. The resulting precipitate was collected by filtration, washed thoroughly with water, and lyophilized to afford the HM-IDPs.

No unexpected or unusually high safety hazards were encountered during the experiments described herein.

Fluorescent spectra measurement

The fluorescence emission spectra of 400 μL solutions for each sample were recorded from 550 nm to 750 nm. All fluorescence emission spectra were obtained using Shimadzu RF-5301PC spectrometer with an excitation wavelength of 410 nm.

Circular dichroism (CD) spectra measurement

CD spectra were recorded (180–300 nm) using a JASCO 8-10 spectrometer under nitrogen atmosphere. Samples were added into a quartz cuvette (Starna 20/C-Q-1, 1 mm pathlength) and scanned with 1 nm interval at the scanning speed of 100 nm/min. Each measurement was accumulated twice, with the Savitzky-Golay smoothing method applied using a convolution width of 13.

UV–vis absorption spectra measurement

UV–vis absorption were recorded (300–700 nm) using a JASCO 8-10 spectrometer under nitrogen atmosphere. Samples were added into a quartz cuvette (1 mm) and scanned with 1 nm interval at the scanning speed of 500 nm/min.

Dynamic Light Scattering (DLS) measurement

DLS measurements were performed using a Wyatt Technology DynaPro Nanostar II instrument at 25 °C. For each measurement, 100 μL of sample was loaded into a cuvette and allowed to equilibrate in the instrument chamber. Scattered light intensity fluctuations were then recorded, with 5 s of data accumulation per measurement. The autocorrelation function was analyzed to obtain the intensity-weighted hydrodynamic size distribution.

Transmission electron microscopy (TEM) sample preparation and imaging

After placing 5 μL samples on 400 mesh copper grids coated with continuous thick carbon film (~35 nm) which was glowed discharged, we washed the grid with ddH2O and UA (uranyl acetate). The sample loaded grid was stained with the UA for 20 seconds. The residual UA was removed by filter paper and then dried in air. TEM images were obtained with FEI Morgagni 268 80 kV with a 1 k × 1 k AMT CCD camera.

Cell culture

Saos2 and HeLa cell line were purchased from American Type Culture Collection (ATCC, USA). Saos2 cells were cultured in McCoy's 5A Medium supplemented with 15% fetal bovine serum (FBS), 1% penicillin streptomycin. HeLa cells were cultured in Minimum Essential Medium (MEM) supplemented with 10% fetal bovine serum (FBS), 1% penicillin streptomycin. Both cell lines were incubated at 37°C in a humidified atmosphere of 5% CO_2.

MTT assay

Cells were seeded in 96-well plates at a density of 1×10^4 cells/well and incubated for 24 hours to allow attachment. After removing the culture medium, fresh culture medium containing different concentration of the precursors were added. After 24, 48, and 72 hours, MTT (ACROS Organics) solution (0.5 mg/mL in culture medium) was added to each well and incubated at 37 °C for 4 hours. Following that, 100 μL of SDS-HCl solution was then added to stop the reduction reaction and dissolve the formazan. The absorbance of each well at 595 nm was measured by a DTX880 Multimode Detector. The results were calculated as the

percentage of cell viability relative to untreated cells. The MTT assay was performed in triplet (n = 3), and the average value of the three measurements was taken.

Confocal laser scanning microscopy (CLSM) Imaging

CLSM images were acquired using a Nikon AX-R ("Dagahra") system. Images were captured with a 60× oil-immersion objective using a resonant bidirectional scanner. Emission was collected from 662-737 nm for the PPIX channel and 420-460 nm for the Hoechst 33342 (nuclei) channel.

Enzymatic Reactions

Proteinase K Treatment: To 200 µL solutions containing PPIX-IDPs in double-distilled water, 2 µL of a 100 mg/mL proteinase K stock solution was added to achieve a final concentration of 1 mg/mL. The reactions were incubated at 37 °C. For controls, 40 µL aliquots were withdrawn from each solution prior to enzyme addition. At designated time points (5 min, 30 min, and 2h), 40 µL aliquots were rapidly frozen in liquid nitrogen to stop the reaction. Samples were then lyophilized at −80 °C, reconstituted in 0.1% TFA in H2O/acetonitrile (1:1 v/v), filtered, and analyzed by LC-MS.

LC-MS analysis was performed with solvent A (water + 0.1% formic acid) and solvent B (acetonitrile + 0.1% formic acid) with a gradient program using the following separation method: 0 min 95%A, 5%B; 1 min 95%A, 5%B; 8 min 1%A, 99%B; 13min 1%A, 99%B; 13.1 min 95%A, 5%B; 14 min 95%A, 5%B. The mass spectrometer was operated in negative ion mode, scanning a range of 50–3000 m/z.

Supporting figures

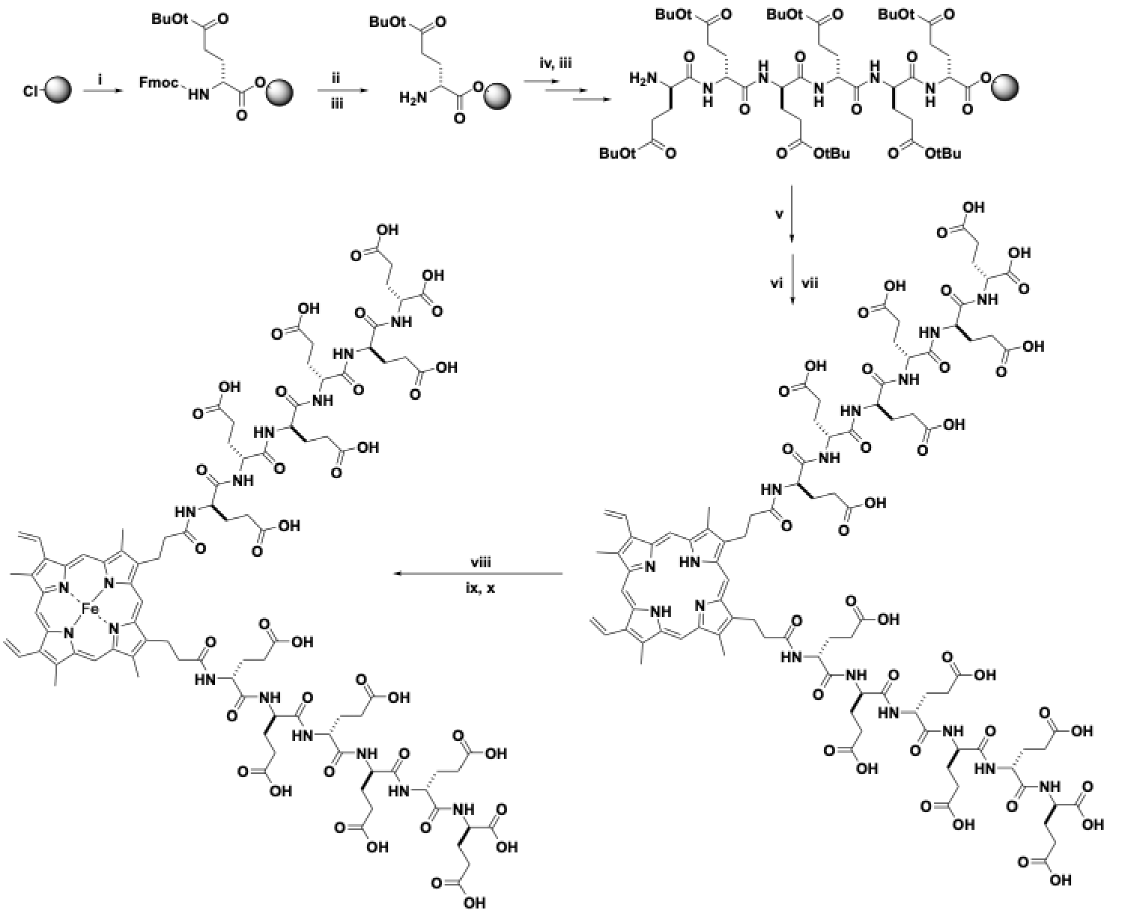

i. 1.0 equiv. Fmoc-D-Glu(OtBu)-OH, 4.0 equiv. DIEA, in DCM, overnight.
ii. DIEA:MeOH:DCM = 1:2:17, 30 min.
iii. 20% piperidine in DMF, 30 min.
iv. 1.0 equiv. Fmoc-D-Glu(OtBu)-OH, 1.0 equiv. HBTU, 1.0 equiv. HOBt, 4.0 equiv. DIEA in DMF, 2 h.
v. 0.5 equiv. PPIX, 1.0 equiv. HBTU, 1.0 equiv. HOBt, 4.0 equiv. DIEA in DMF, overnight.
vi. 99% TFA, 1 h.
vii. Air-dry TFA, precipitate with cold ethyl ether, filter, collect solid crude.
viii. 50 equiv. $FeCl_2$ in DMF, 110 °C, 24 h.
ix. Rotavap DMF, add 1 M NaOH, adjust to pH 9, keep filtrate.
x. Add 1 M HCl, adjust to pH 2, 2 h, filter, collect solid.

Scheme S1. Synthetic route for HM-$(e_6)_2$.

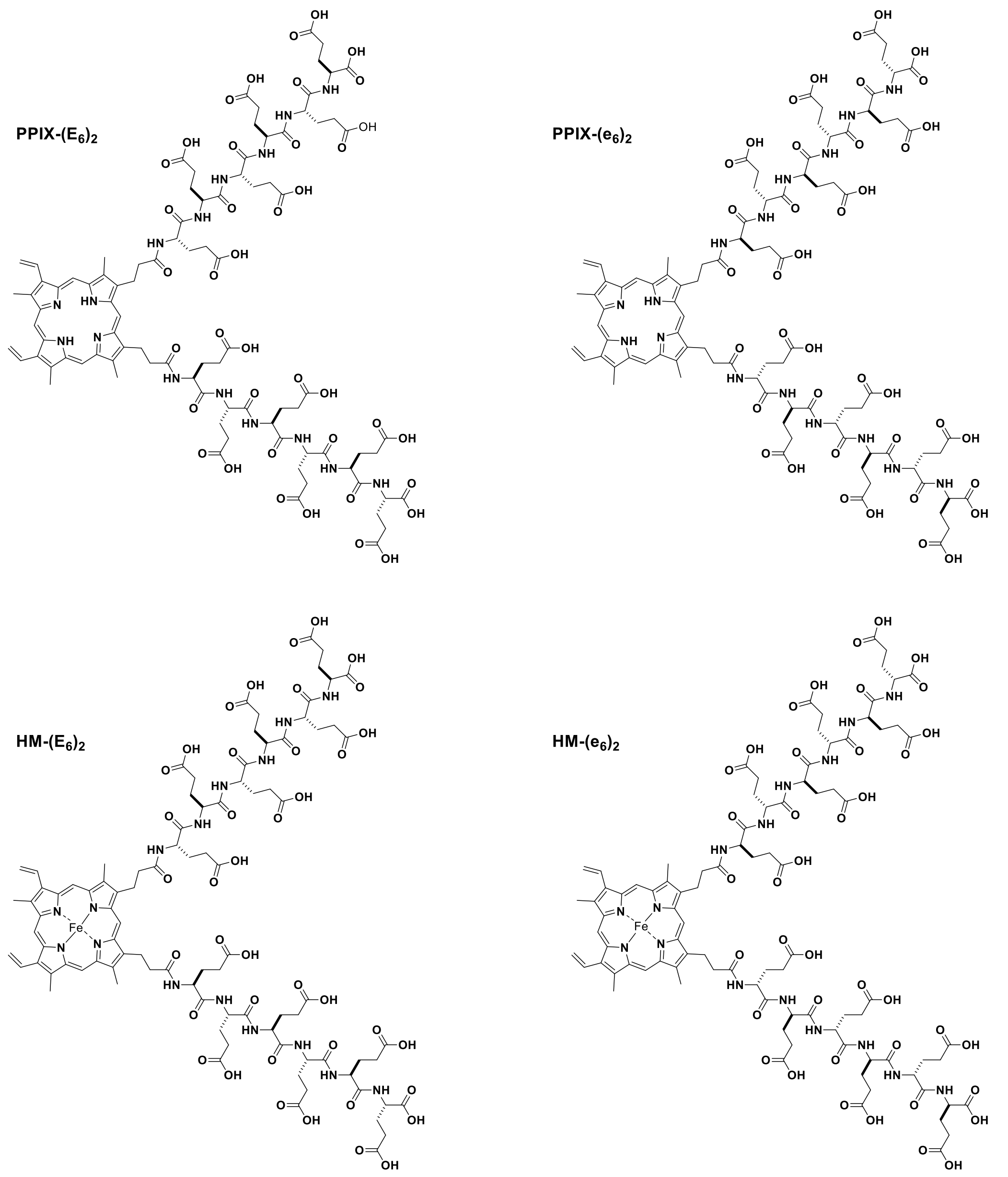

Figure S1. Chemical strucuture of PPIX-(E_6)$_2$, PPIX-(e_6)$_2$, HM-(E_6)$_2$, and HM-(e_6)$_2$.

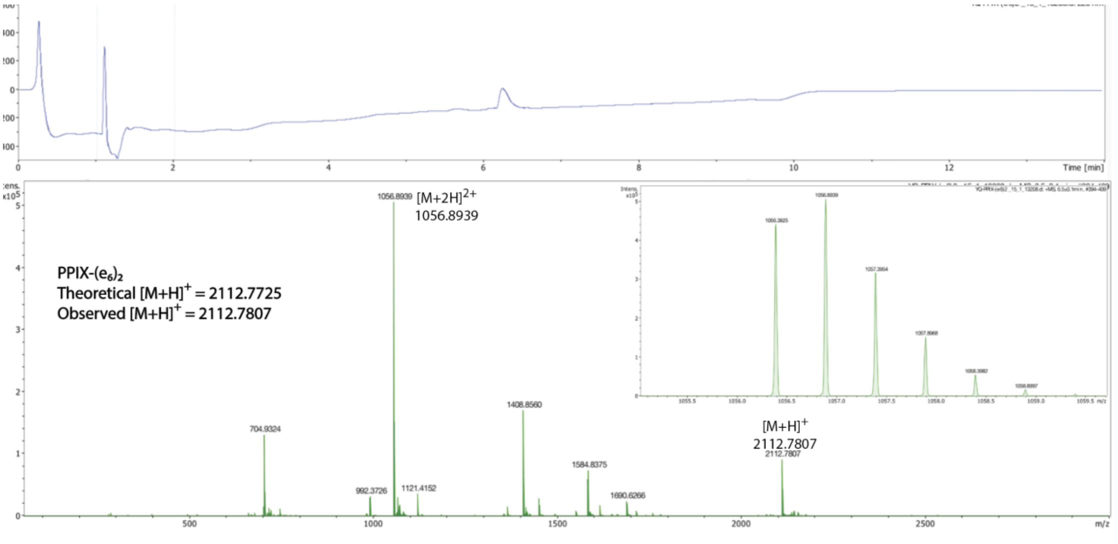

Figure S2. LC-MS spectrum of PPIX-(e_6)$_2$.

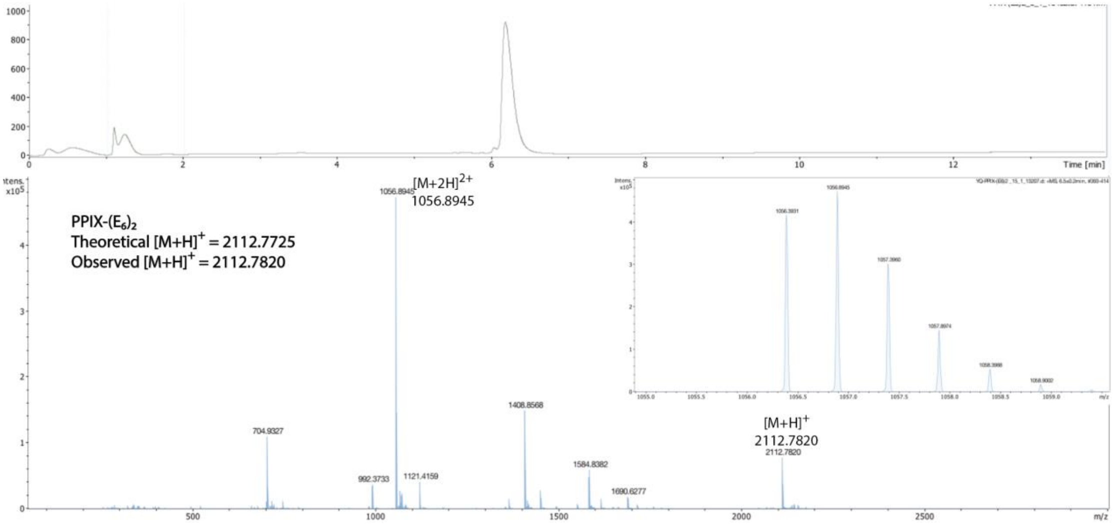

Figure S3. LC-MS spectrum of PPIX-(E_6)$_2$.

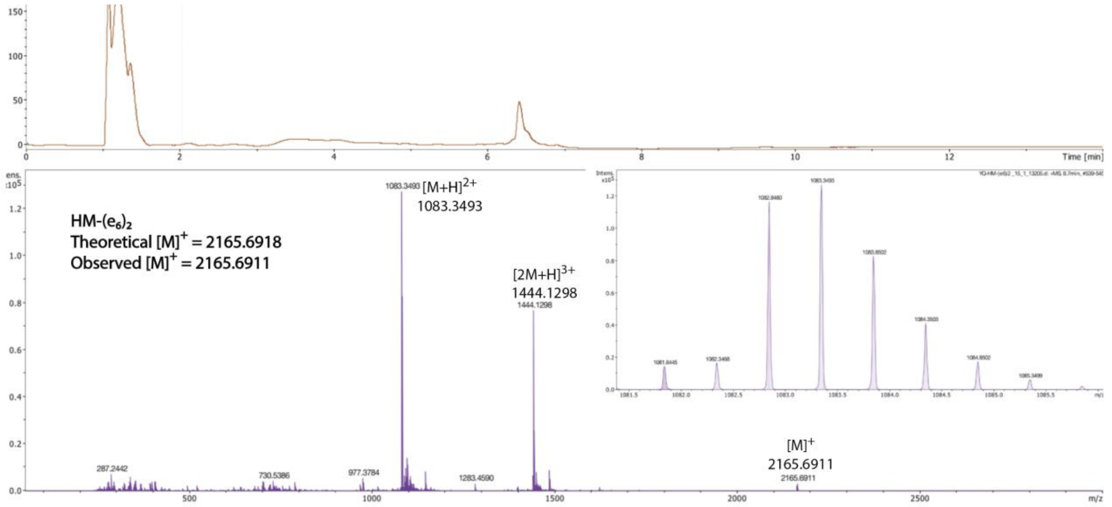

Figure S4. LC-MS spectrum of HM-(e_6)$_2$.

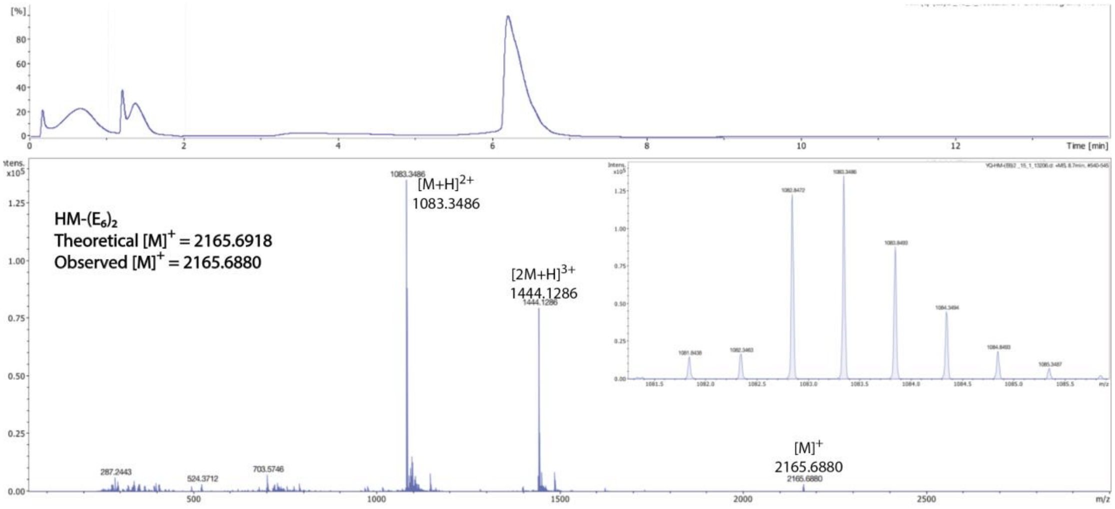

Figure S5. LC-MS spectrum of HM-(E_6)$_2$.

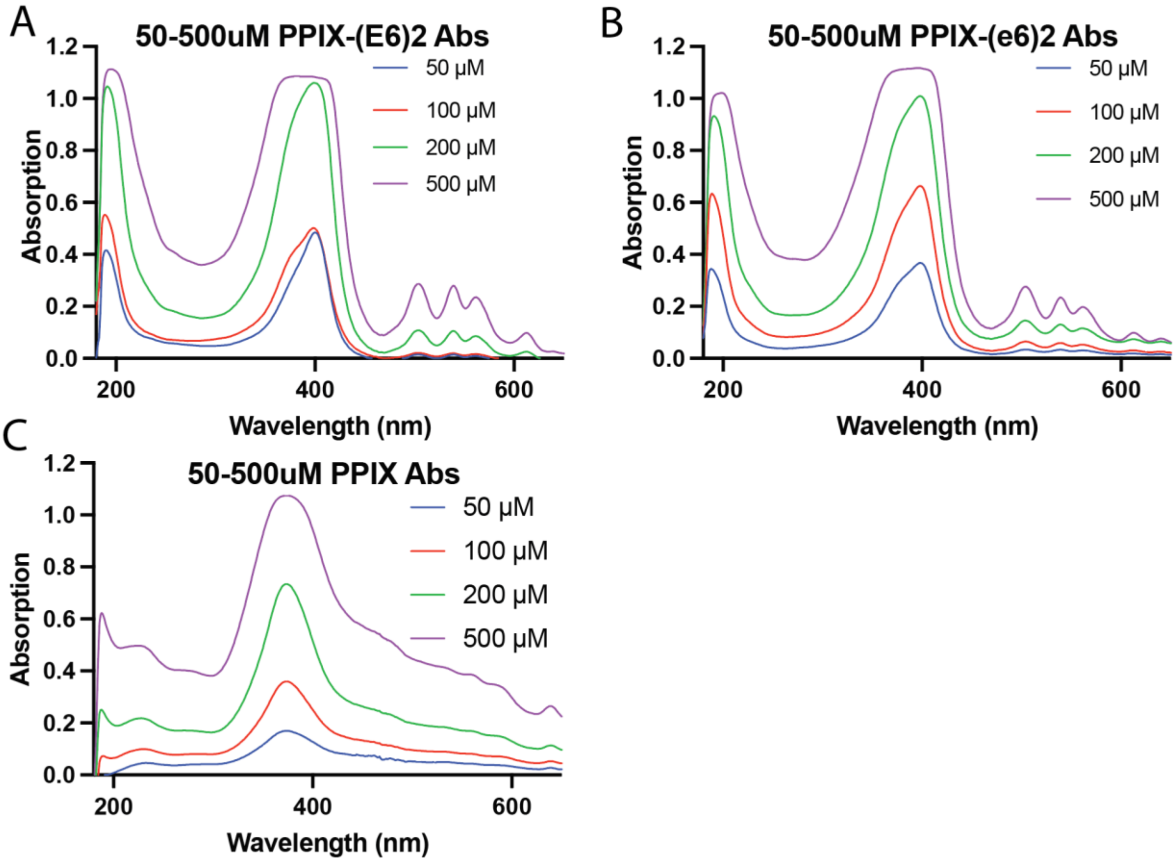

Figure S6. UV–vis absorption spectra of (A) PPIX-$(E_6)_2$, (B) PPIX-$(e_6)_2$, and (C) PPIX at concentrations ranging from 50 to 500 μM in aqueous solution (pH 7).

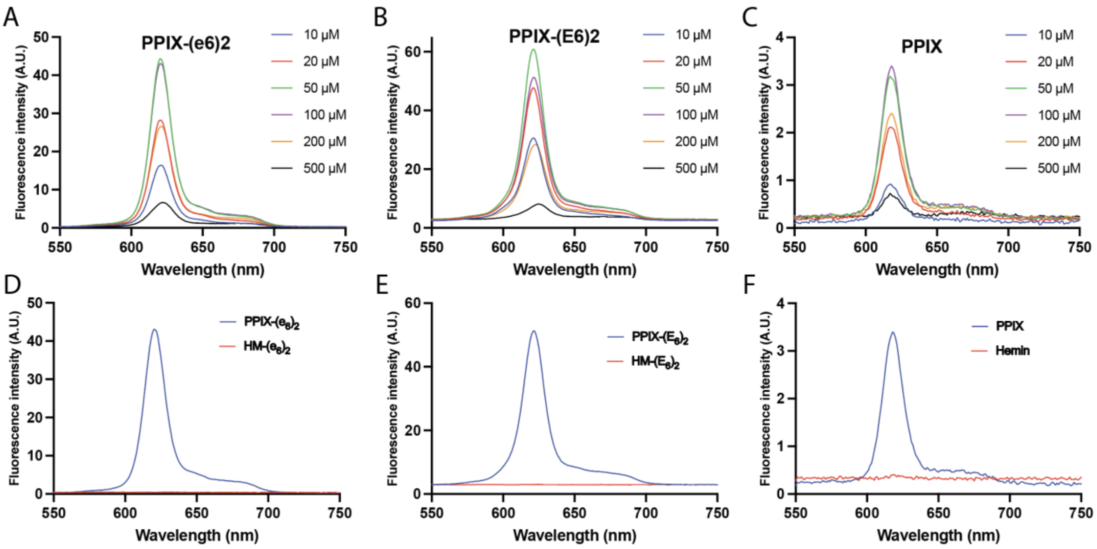

Figure S7. Fluorescence emission spectra of (A) PPIX-$(e_6)_2$, (B) PPIX-$(E_6)_2$, and (C) PPIX at concentrations ranging from 10 to 500 μM and 100 μM (D) PPIX-$(e_6)_2$ and HM-$(e_6)_2$, (E) PPIX-$(E_6)_2$ and HM-$(E_6)_2$, and (F) PPIX and hemin in aqueous solution at pH 7.

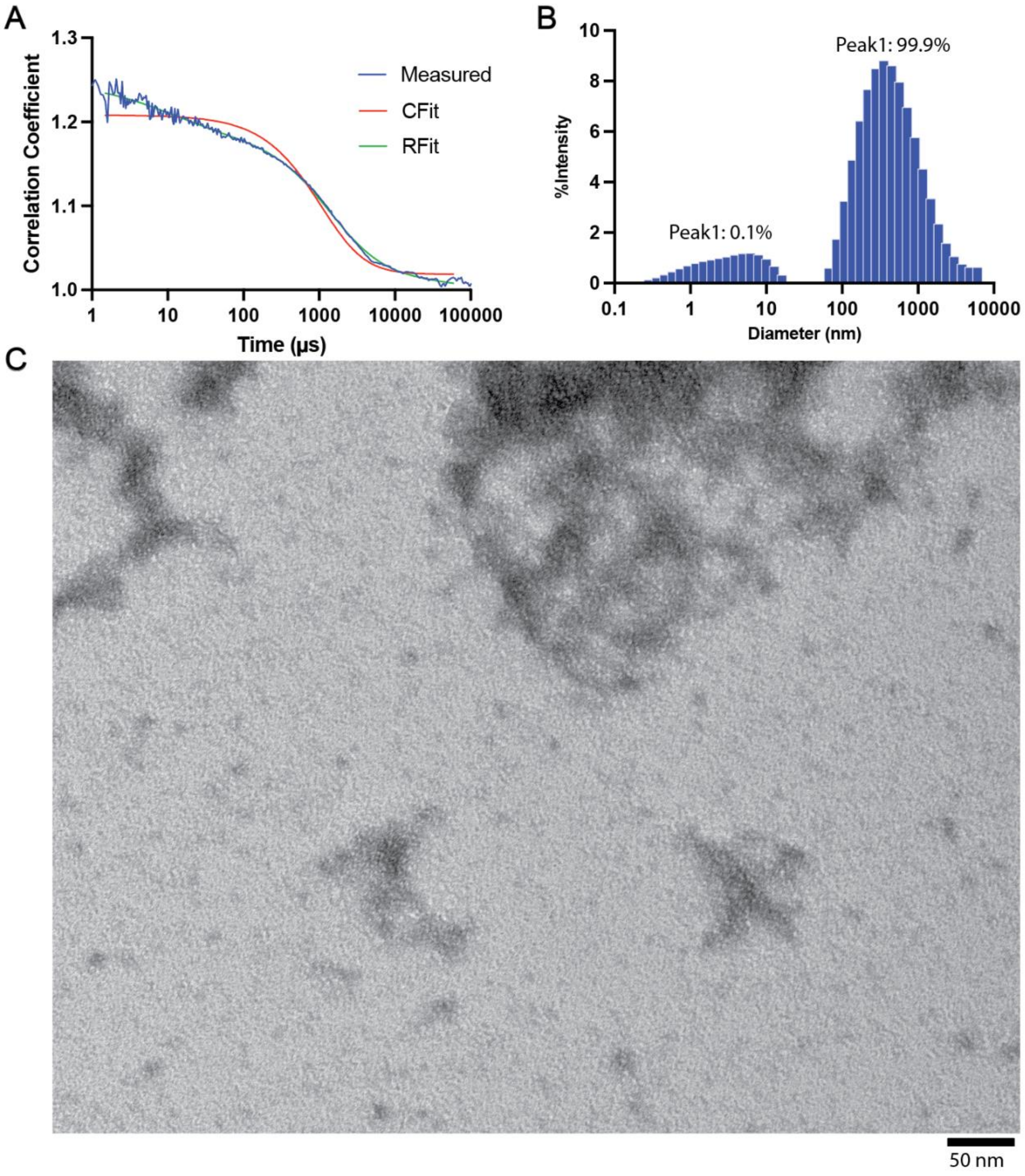

Figure S8.(A) Correlation function (blue: measured; red: cumulant fit; green: regularization fit), (B) size distribution by intensity, and (C) TEM images of 25 µM HM-$(e_6)_2$.

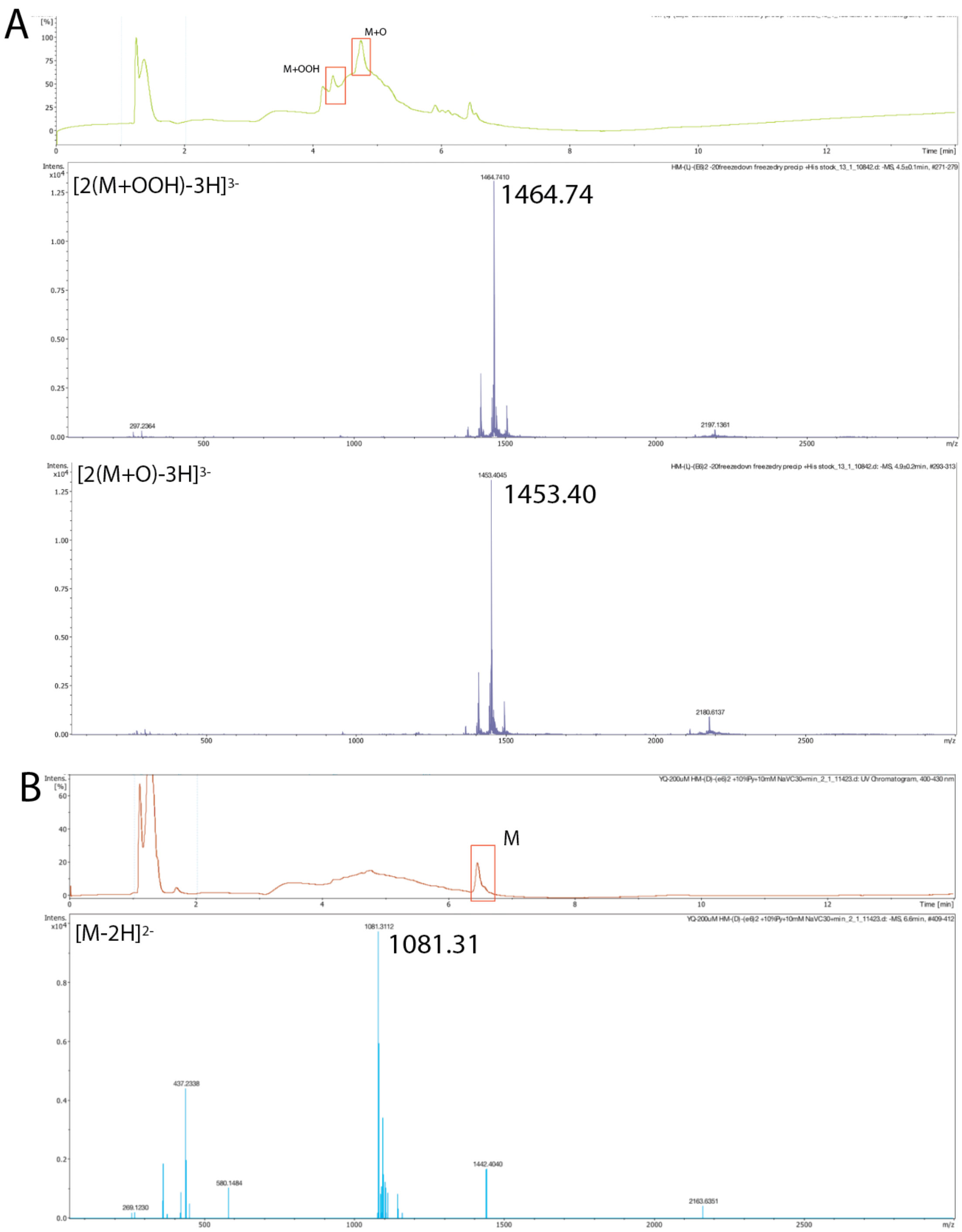

Figure S9. LC–MS spectrum of (A) lyophilized HM-$(e_6)_2$ redissolved in water. (B) 200 μM HM-$(e_6)_2$ dissolved in 1 M pyridine in water and treated with 10 mM vitamin C (VC) for 30 min.

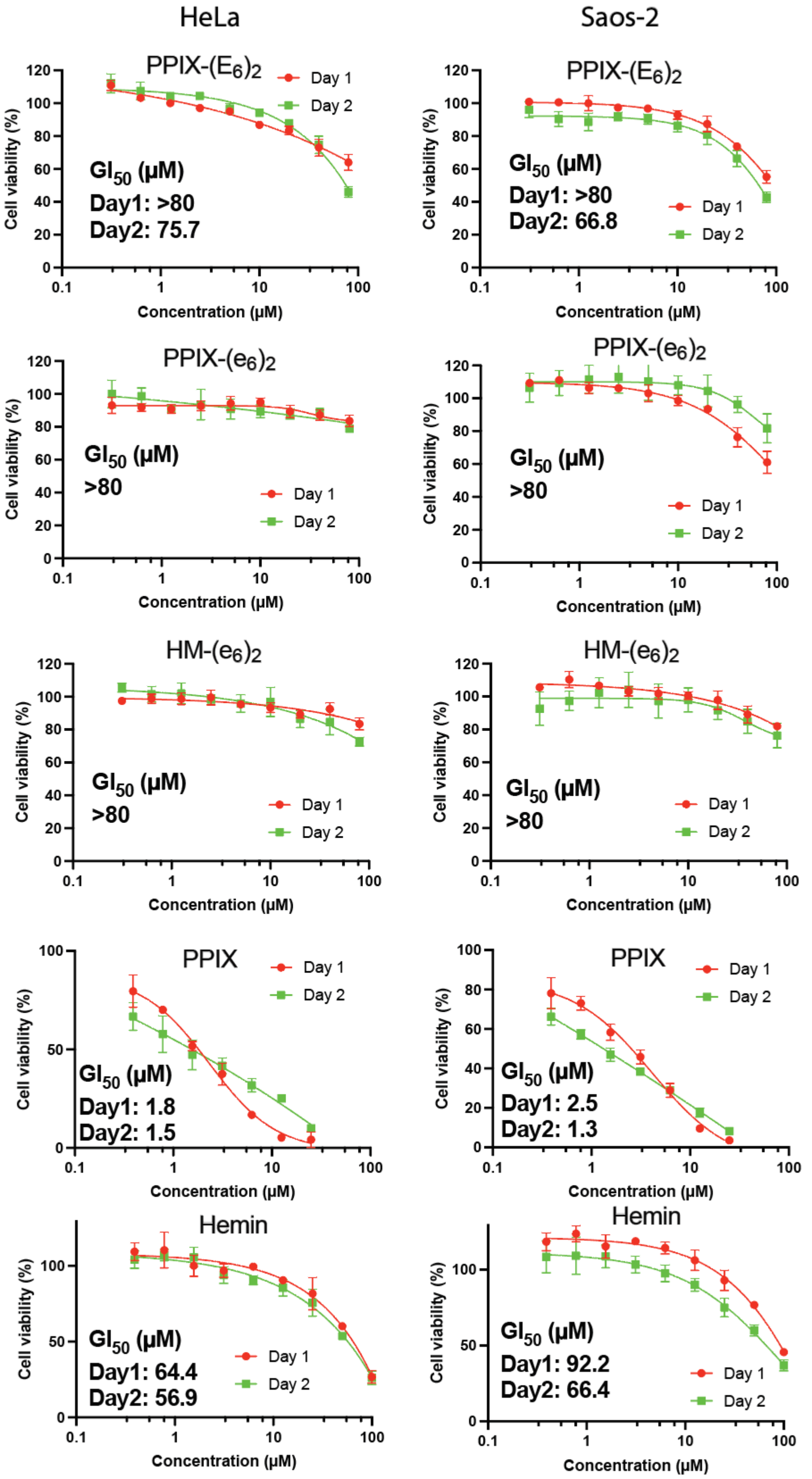

Figure S10. Cytotoxicity of PPIX-$(E_6)_2$, PPIX-$(e_6)_2$, HM-$(e_6)_2$, PPIX, and hemin in Saos-2 and HeLa cells after 2 days of treatment.

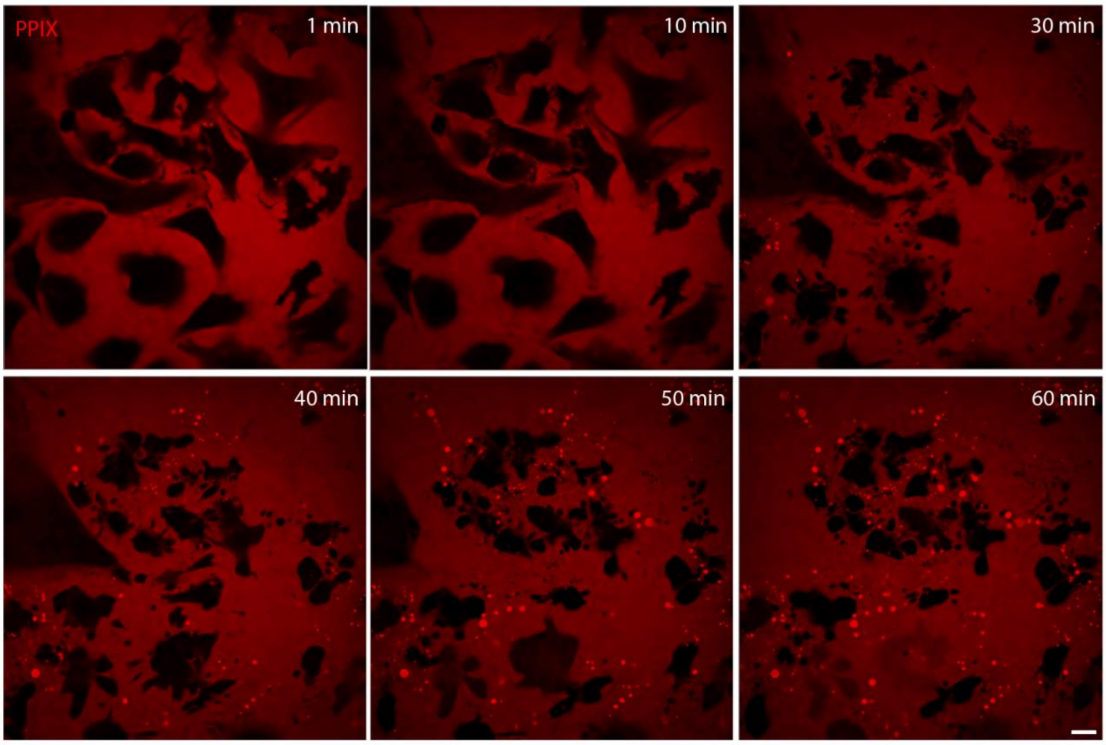

Figure S11. Time-lapse CLSM images of HeLa cells co-incubated with 50 µM PPIX-$(e_6)_2$ for 1 hour. (Scale bar = 20 µm. Contrast adjusted by ImageJ)

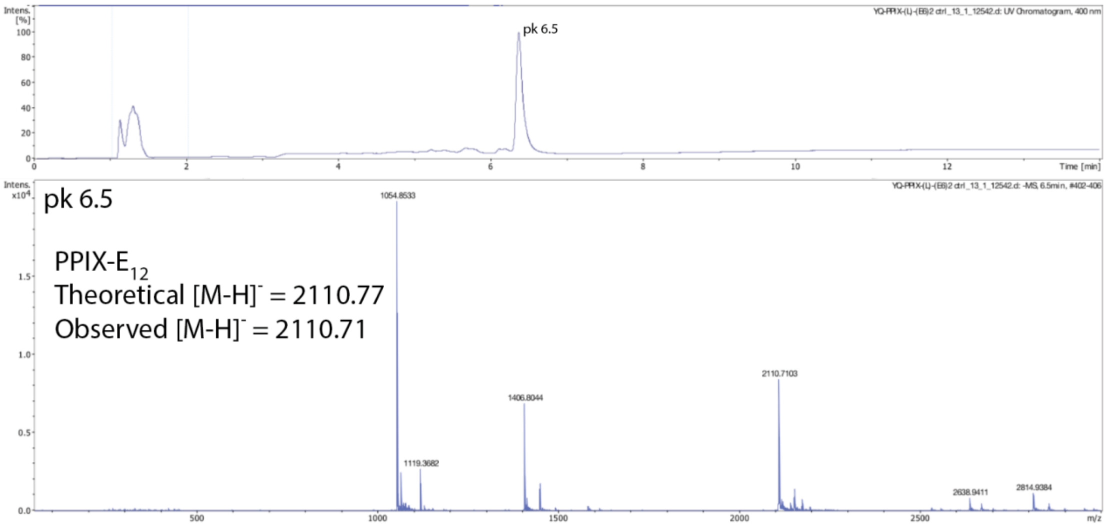

Figure S12. LC–MS analysis of PPIX-$(E_6)_2$ prior to treatment with proteinase K.

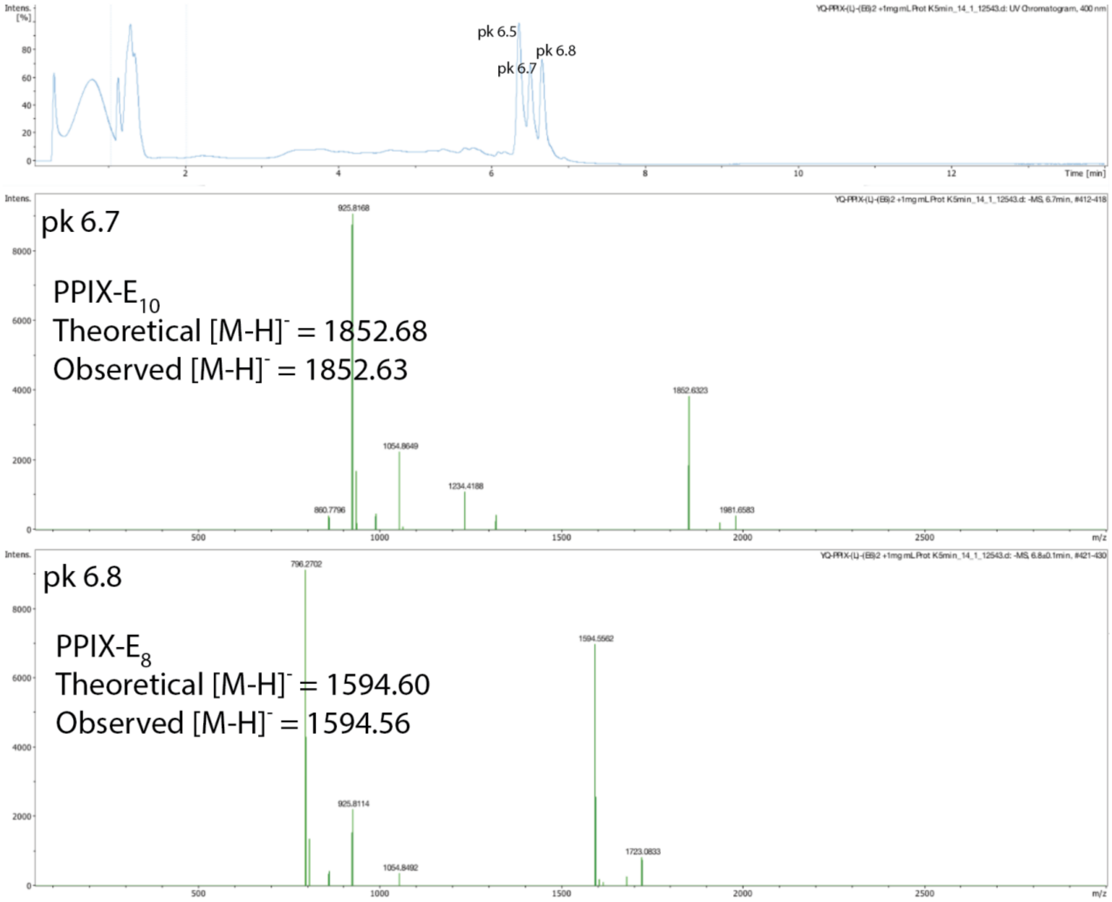

Figure S13. LC–MS analysis of PPIX-$(E_6)_2$ after treatment with 1 mg/mL proteinase K for 5 min.

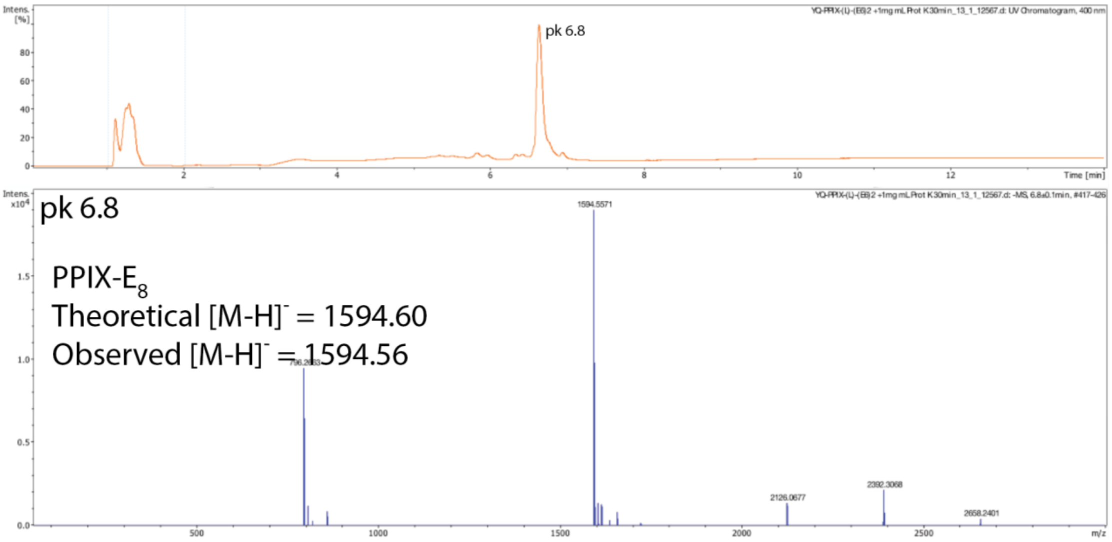

Figure S14. LC–MS analysis of PPIX-$(E_6)_2$ after treatment with 1 mg/mL proteinase K for 30 min.

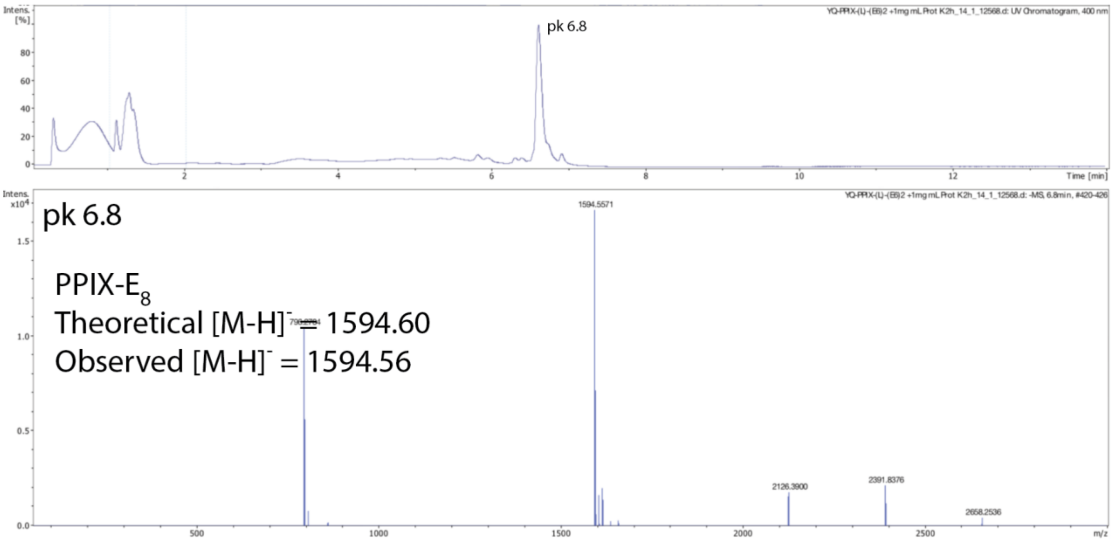

Figure S15. LC–MS analysis of PPIX-$(E_6)_2$ after treatment with 1 mg/mL proteinase K for 2h.

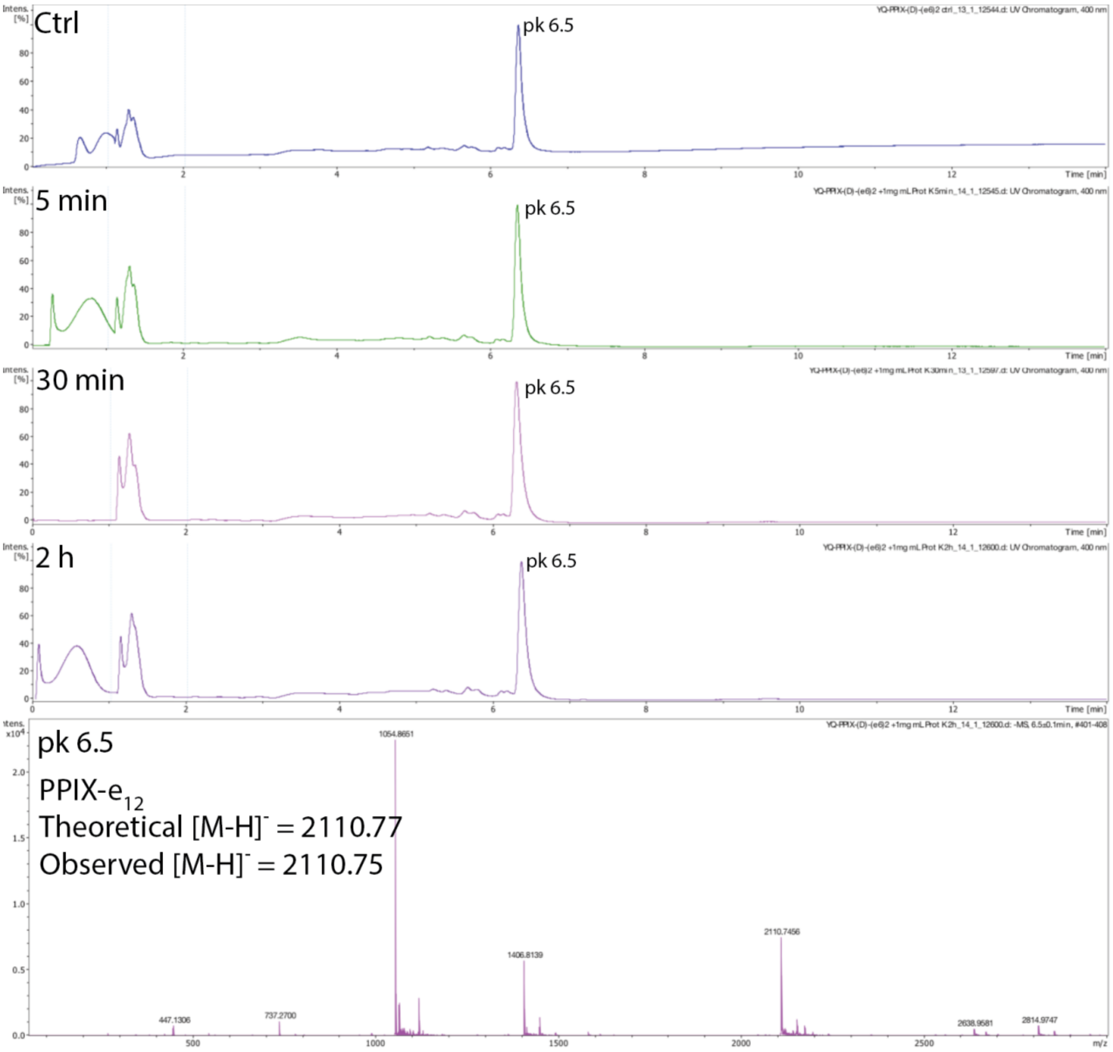

Figure S16. LC–MS analysis of PPIX-$(e_6)_2$ before treatment (control) and after incubation with 1 mg/mL proteinase K for 5 min, 30 min, and 2 h.

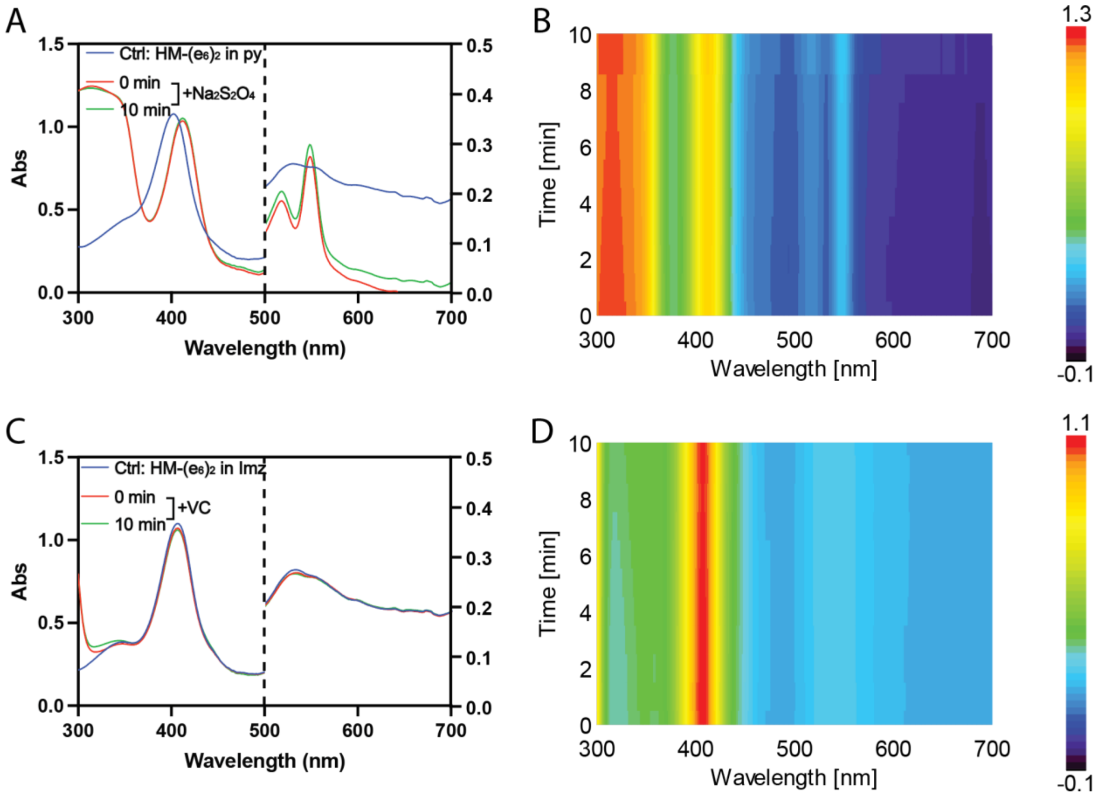

Figure S17. (A) UV–vis absorption spectra of 200 μM HM-$(e_6)_2$ in 1 M pyridine (control) and after addition of 10 mM $Na_2S_2O_4$ at 0 min and 10 min. (B) Corresponding heatmap showing spectral changes over 10 min. (C) UV–vis absorption spectra of 200 μM HM-$(e_6)_2$ in 1 M imidazole (control) and after addition of 10 mM vitamin C (VC) at 0 min and 10 min. (D) Corresponding heatmap showing spectral changes over 10 min.

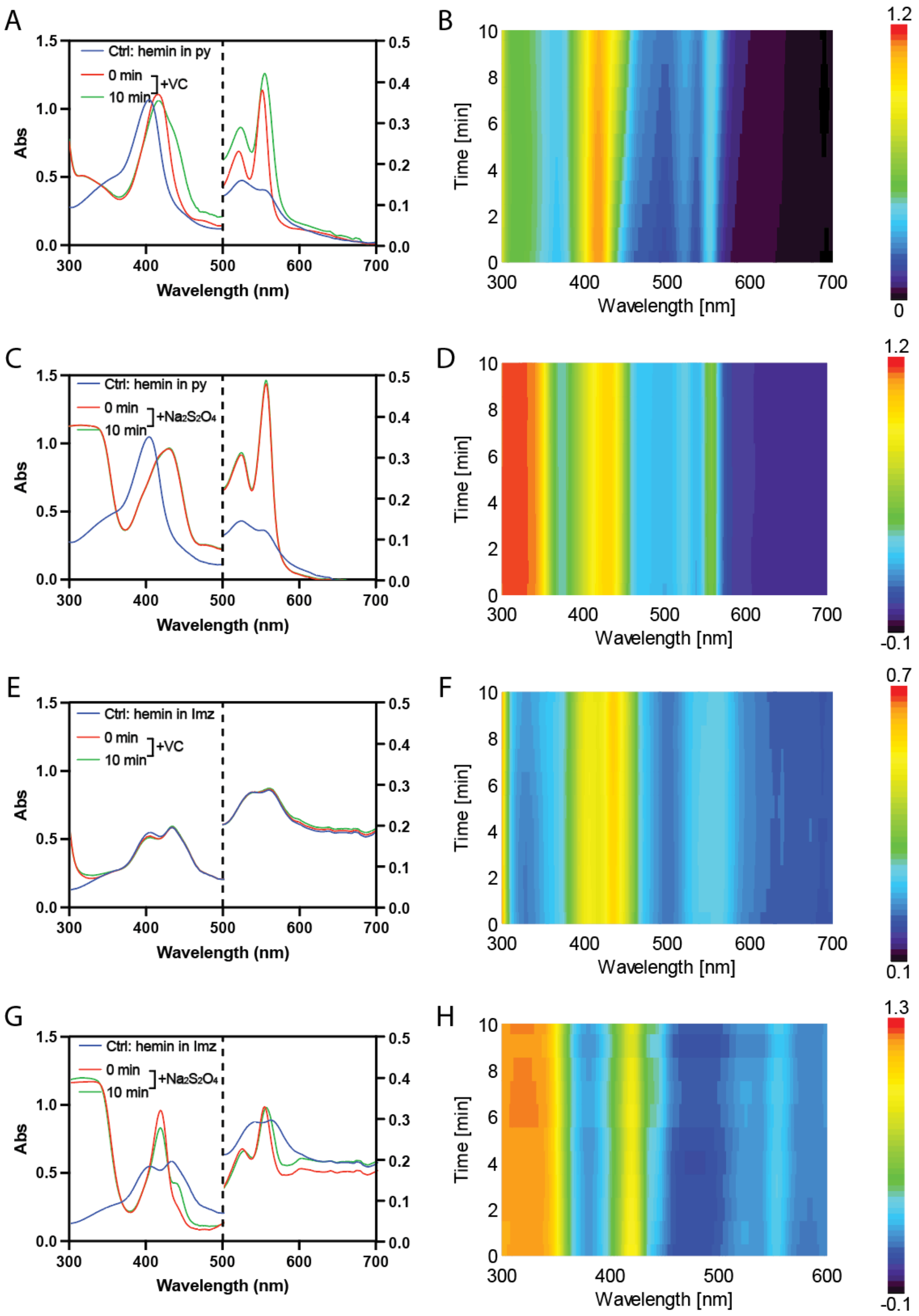

Figure S18. (A) UV–vis absorption spectra of 200 μM hemin in 1 M pyridine (control) and after addition of 10 mM vitamin C (VC) at 0 min and 10 min. (B) Corresponding heatmap showing spectral changes over 10 min. (C) UV–vis absorption spectra of 200 μM hemin in 1 M imidazole (control) and after addition of 10 mM $Na_2S_2O_4$ at 0 min and 10 min. (D) Corresponding heatmap showing spectral changes over 10 min. (E) UV–vis absorption spectra of 200 μM hemin in 1 M pyridine (control) and after addition of 10 mM $Na_2S_2O_4$ at 0 min and 10 min. (F) Corresponding heatmap showing spectral changes over 10 min. (G) UV–vis absorption spectra of 200 μM hemin in 1 M imidazole (control) and after addition of 10 mM vitamin C (VC) at 0 min and 10 min. (H) Corresponding heatmap showing spectral changes over 10 min.

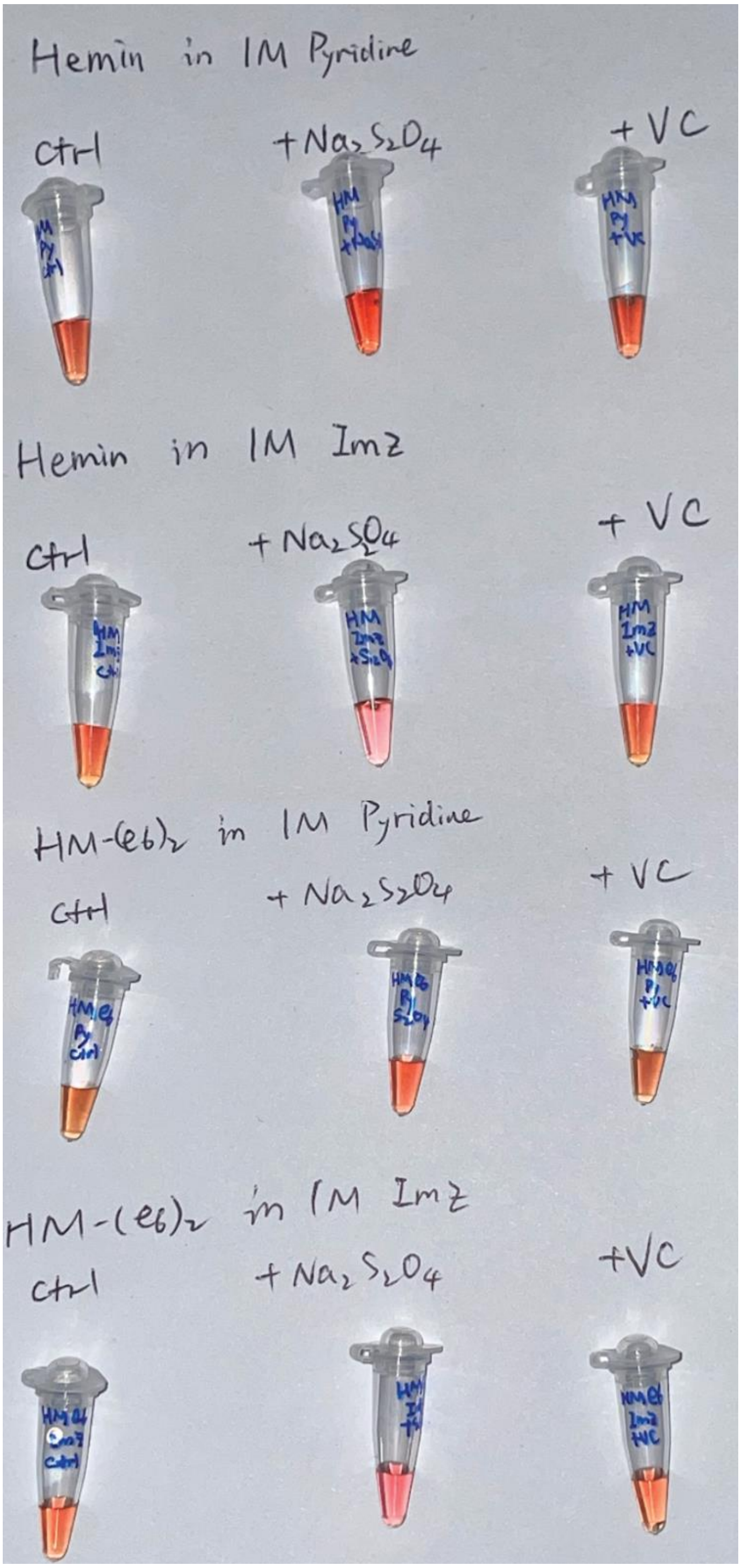

Figure S19. Photographs of 200 μM hemin or HM-(e_6)$_2$ in 1 M imidazole or 1 M pyridine (control) and after treatment with 10 mM $Na_2S_2O_4$ or vitamin C for 1 min.

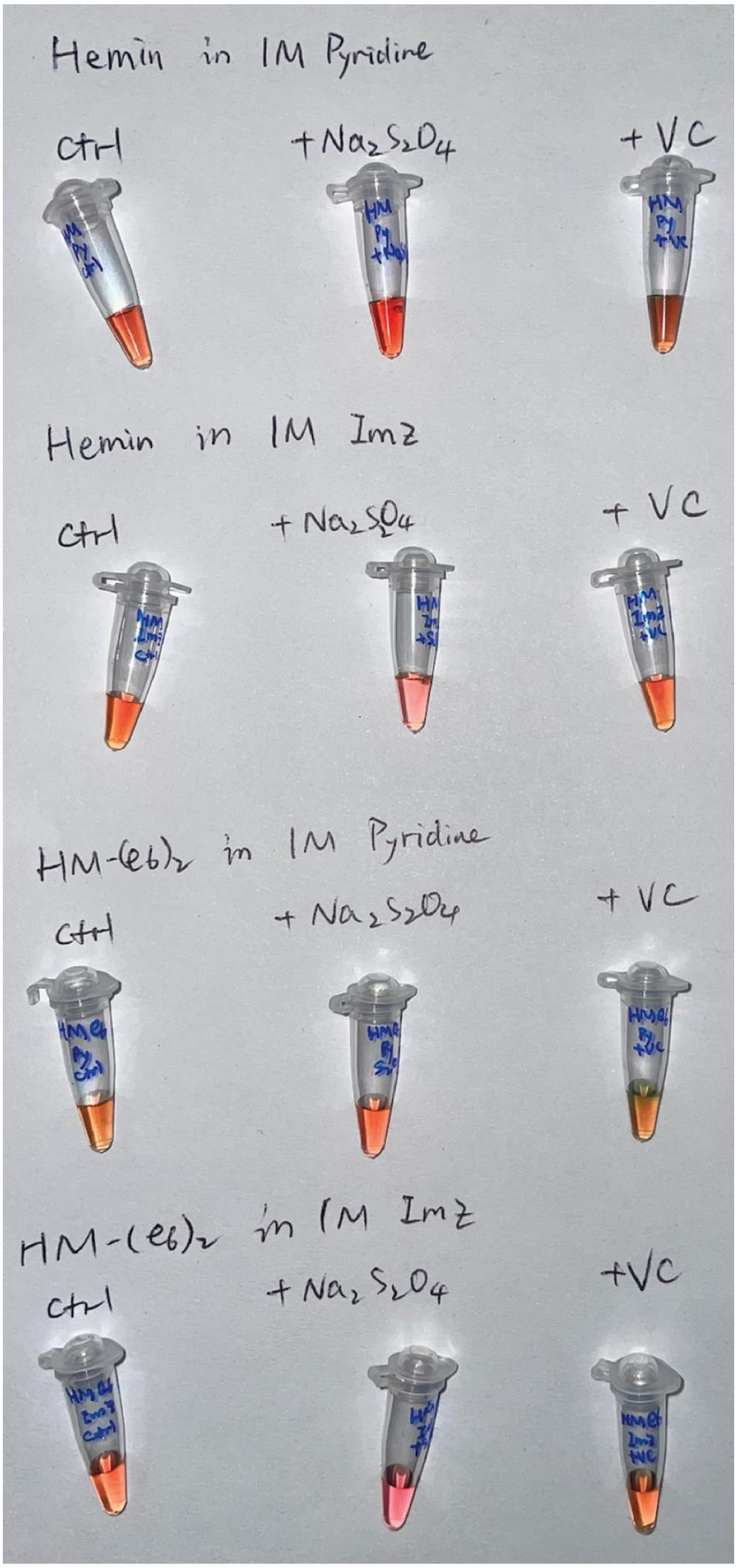

Figure S20. Photographs of 200 μM hemin or HM-$(e_6)_2$ in 1 M imidazole or 1 M pyridine (control) and after treatment with 10 mM $Na_2S_2O_4$ or vitamin C for 10 min.
